# Plasmid-free production of the plant lignan pinoresinol in growing *Escherichia coli* cells

**DOI:** 10.1186/s12934-024-02562-3

**Published:** 2024-10-23

**Authors:** U. Joost Luelf, Alexander Wassing, Lisa M. Böhmer, Vlada B. Urlacher

**Affiliations:** Institute of Biochemistry, Heinrich Heine University Düsseldorf, 40225 Düsseldorf, Germany

**Keywords:** Phenylpropanoid, Coniferyl alcohol, Ferulic acid, Lignan, Pinoresinol, Oxidative coupling, *E. coli*, Chromosome, CRISPR/Cas

## Abstract

**Background:**

The high-value aryl tetralin lignan (+)-pinoresinol is the main precursor of many plant lignans including (-)-podophyllotoxin, which is used for the synthesis of chemotherapeutics. As (-)-podophyllotoxin is traditionally isolated from endangered and therefore limited natural sources, there is a particular need for biotechnological production. Recently, we developed a reconstituted biosynthetic pathway from (+)-pinoresinol to (-)-deoxypodophyllotoxin, the direct precursor of (-)-podophyllotoxin, in the recombinant host *Escherichia coli*. However, the use of the expensive substrate (+)-pinoresinol limits its application from the economic viewpoint. In addition, the simultaneous expression of multiple heterologous genes from different plasmids for a multi-enzyme cascade can be challenging and limits large-scale use.

**Results:**

In this study, recombinant plasmid-free *E. coli* strains for the multi-step synthesis of pinoresinol from ferulic acid were constructed. To this end, a simple and versatile plasmid toolbox for CRISPR/Cas9-assisted chromosomal integration has been developed, which allows the easy transfer of genes from the pET vector series into the *E. coli* chromosome. Two versions of the developed toolbox enable the efficient integration of either one or two genes into intergenic high expression loci in both *E. coli* K-12 and B strains. After evaluation of this toolbox using the fluorescent reporter mCherry, genes from *Petroselinum crispum* and *Zea mays* for the synthesis of the monolignol coniferyl alcohol were integrated into different *E. coli* strains. The product titers achieved with plasmid-free *E. coli* W3110(T7) were comparable to those of the plasmid-based expression system. For the subsequent oxidative coupling of coniferyl alcohol to pinoresinol, a laccase from *Corynebacterium glutamicum* was selected. Testing of different culture media as well as optimization of gene copy number and copper availability for laccase activity resulted in the synthesis of 100 mg/L pinoresinol using growing *E. coli* cells.

**Conclusions:**

For efficient and simple transfer of genes from pET vectors into the *E. coli* chromosome, an easy-to-handle molecular toolbox was developed and successfully tested on several *E. coli* strains. By combining heterologous and endogenous enzymes of the host, a plasmid-free recombinant *E. coli* growing cell system has been established that enables the synthesis of the key lignan pinoresinol.

**Supplementary Information:**

The online version contains supplementary material available at 10.1186/s12934-024-02562-3.

## Background

**Scheme 1:**
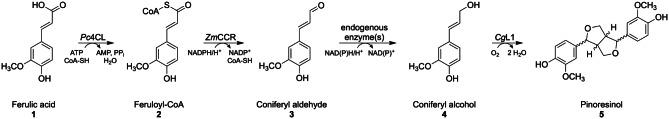
Recombinant biosynthetic path from ferulic acid **1** to pinoresinol **5** in *E. coli*. The substrate is sequentially converted by a 4-coumarate-CoA ligase from *P. crispum* (*Pc*4CL), a cinnamoyl-CoA reductase from *Z. mays* (*Zm*CCR), *E. coli* alcohol dehydrogenases or aldo-keto reductases and a laccase from *C. glutamicum* (*Cg*L1)

Lignans are a large and structurally diverse group of pharmacologically active secondary plant metabolites which are formed via oxidative dimerization of phenylpropanoids [1, 2]. In mammals, dietary intake of lignans from plants or plant-derived foods leads to the formation of enterolignans by the gut microbiome, which are classified as phytoestrogens and show health-promoting effects [3]. Furthermore, the chemotherapeutic drugs etoposide and teniposide are semi-synthetically produced from the aryl tetralin lignan (-)-podophyllotoxin which is isolated from the endangered plant *Podophyllum hexandrum* Royle [4]. The low content of lignans in plants combined with a high demand and their structural complexity leads to a particular need for biotechnological solutions to produce these natural products [5, 6]. The biosynthesis starts from cinnamic acid derivatives which originate from the shikimate and phenylpropanoid pathways. Depending on the phenylpropanoid monomer, different lignans are formed. The major subclass of furofuran lignans, including the title compound pinoresinol **5**, results from the dimerization of the monolignol coniferyl alcohol **4** [1]. Besides being a starting precursor for higher lignans, pinoresinol **5** possesses multiple biological activities including anti-inflammatory [7] and antioxidant activity, which also explains its hepatoprotective [8] and chemopreventive [9] properties. Furthermore, antifungal [10] and putative hypoglycemic properties [11] add to the list of pharmacological activities of interest. The above-mentioned chemotherapeutics etoposide and teniposide are derived from pinoresinol **5** via (-)-podophyllotoxin, indicating potentially high demand of this high-value compound. A first step towards the biotechnological production of these drugs in recombinant microorganisms was recently taken by our group by synthesizing (-)-deoxypodophyllotoxin from (+)-pinoresinol in the heterologous host *Escherichia coli* [12]. However, the use of the high-value compound (+)-pinoresinol as a starting material remains an economic challenge. (+)-Pinoresinol has been isolated from plants with high enantiopurity but low efficiency [13]. As the subsequent reactions from pinoresinol **5** towards (-)-deoxypodophyllotoxin are catalyzed by enantioselective enzymes, racemic pinoresinol **5** instead of (+)-pinoresinol could be used as starting substrate [14, 15]. Thus, in this study, we engineered and optimized growing *E. coli* cells for the production of racemic pinoresinol **5**. Starting from relatively inexpensive and readily available ferulic acid **1**, we reconstituted an artificial phenylpropanoid path in *E. coli* to first produce the monolignol coniferyl alcohol **4**. Ferulic acid **1** is reduced to coniferyl alcohol **4** via coniferyl aldehyde **3** in three enzymatic steps (Scheme 1). Two of the three steps are catalyzed by enzymes derived from the plants *Petroselinum crispum* and *Zea mays* [16, 17]. The final reduction step is catalyzed by endogenous *E. coli* alcohol dehydrogenases or aldo-keto reductases [18, 19]. For the synthesis of pinoresinol **5** from **4**, laccases [20–22] and peroxidases [23] have been successfully applied in the past. Previously, we have demonstrated that a laccase from *Corynebacterium glutamicum* gave the highest pinoresinol concentration in vitro and thus was used in this study [21]. In the reported reactions with isolated enzymes or in resting *E. coli* cells, pinoresinol concentrations ranged between several milligrams to grams per liter, depending on the complexity of the system, the starting substrate molecule, its concentration, and the number of reaction steps [14, 21–24].

In order to simplify the bioprocess design, growing cells were preferred instead of resting cells used for the production of (-)-deoxypodophyllotoxin [12] and its precursors [15] in our previous studies. In addition to the facts that enzymes are steadily produced during the reactions and that only one reaction vessel is required, the combination of cell growth and substrate conversion eliminates the need to prepare the cells for a resting cell approach and is therefore significantly less time-consuming.

Furthermore, plasmids were used for the co-expression of all genes in our previous work. Plasmids offer many advantages like easy cloning, fast and efficient transformation of different host strains, and high and tunable expression levels. However, there are also some shortcomings. As plasmids are extrachromosomal, non-essential DNA, a form of selectable markers has to be used to maintain the plasmids in the host cell and prevent plasmid loss. For this purpose, antibiotics are often utilized, which are cost-intensive on industrial scale, can affect the cell growth, and contribute to the development of multidrug-resistant human pathogens [25–28]. In recombinant strains with plasmids, the number of gene copies can vary from cell to cell even within stable populations, leading to altered expression levels [25, 28]. It should also be noted that the plasmid copy number can increase drastically after induction resulting in an increased metabolic burden [25, 27]. The transition from plasmid-based to chromosomal expression helps to avoid most of these issues. To this end, we developed an easy-to-handle toolbox that allows the simple transfer of genes from the often-used pET vector series into the *E. coli* chromosome based on CRISPR/Cas-assisted λ-Red-mediated homologous recombination [29, 30].

Since the expression levels of chromosomally integrated genes depend on their position in the genome, different integration sites can be used to tune expression levels. One general rule is the so-called ‘gene dosage effect’, which states that genes located closer to the chromosome’s origin of replication (*oriC*) are statistically more abundant in the cell and therefore more highly expressed [31, 32]. In addition, there are regional effects that affect the transcription of integrated genes, resulting in expression levels that vary on average by 2-4-fold depending on the integration site, with rare outliers of ~ 300-fold deviation [31, 33, 34]. To achieve the highest possible integration efficiencies and expression levels, several previously uncharacterized integration loci present in both *E. coli* K-12 and B strains were first identified and analyzed using the fluorescent reporter mCherry.

Further, we aimed to apply a method that does not require the separate synthesis of linear donor DNA but instead allows the use of the same type II restriction enzymes as used for the cloning of pET vectors. The use of circular instead of linear donor DNA for the CRISPR/Cas-based recombination circumvents potential degradation by exonucleases [35]. In addition, the transformation of supercoiled plasmids is highly efficient and high-fidelity replication within the cell results in a higher intracellular repair template concentration compared to linear PCR-synthesized donor DNA [36]. The combination of easy cloning and characterized integration loci in both *E. coli* K-12 and B strains aims to address the biocatalysis community that already has pET-based libraries in hand and wants to take the step towards plasmid-free whole-cell biocatalysis.

In this study, the application of the developed toolbox led to a highly productive whole-cell system for pinoresinol **5** synthesis which was finally obtained after testing *E. coli* K-12 and B strains and optimizing culture media, gene copy number, and copper availability for laccase activity.

## Results and discussion

### Design of pgRNA-ET and pgRNA-DUET vector series for chromosomal integration

**Fig. 1:**
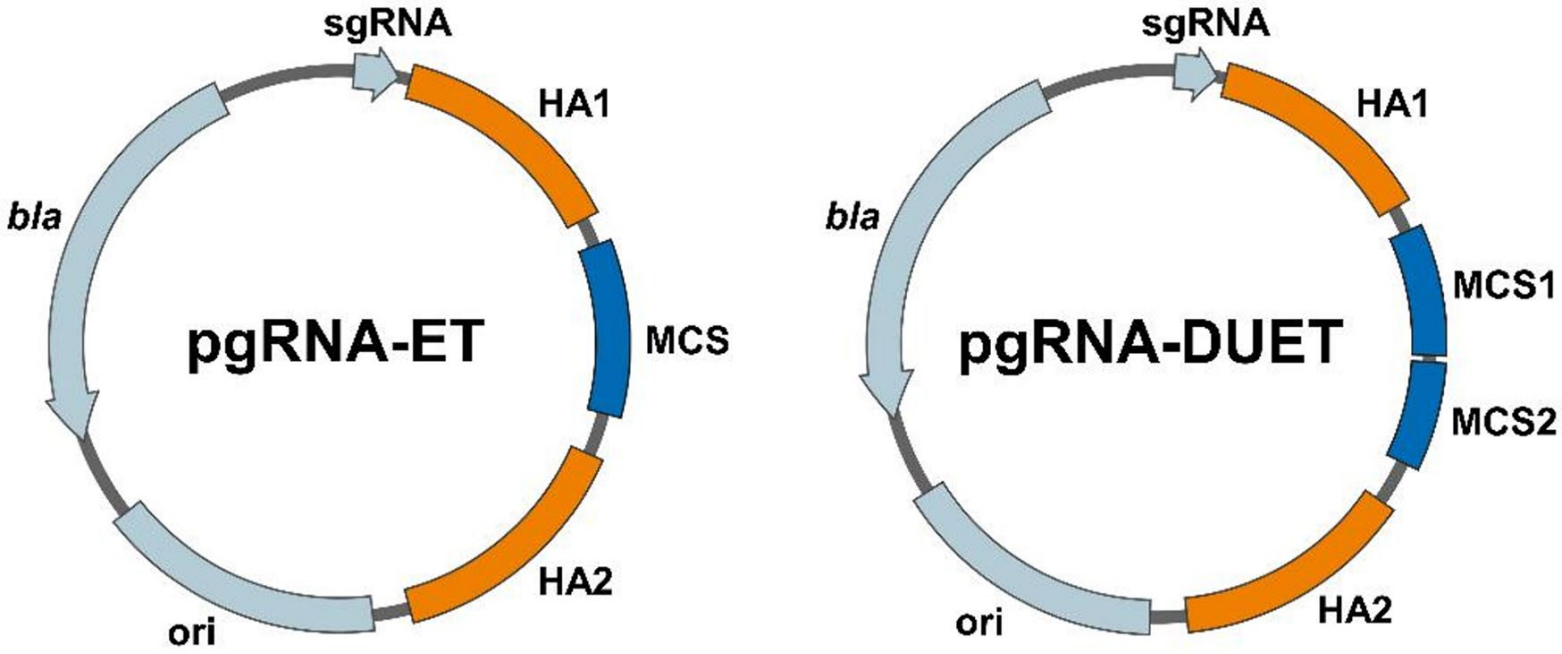
Plasmid series pgRNA-ET (left) and pgRNA-DUET (right) for easy CRISPR/Cas-assisted transfer of genes from pET vectors into the *E. coli* chromosome. Each vector contains a pBR322 origin of replication (ori), a β-lactamase encoding gene (*bla*), a locus-specific single-guide RNA (sgRNA) as well as the corresponding homology arms (HA1 & HA2) for homologous recombination, and multiple cloning sites originating from pET-28a(+) or pETDuet-1 for pgRNAET and pgRNA-DUET, respectively

First, we identified several new intergenic integration sites present in both *E. coli* K-12 and B strains. To this end, information on the prototype *E. coli* K-12 strain MG1655 from its GenBank genome file (accession no. U00096.3), the information in RegulonDB [37] for promoter and terminator sets of this strain, and EcoCyc database [38] for detailed operon information, were combined. Additionally, a study by Vora et al. [39], who described the protein landscape of the genome and identified so-called *highly-expressed extended protein occupancy domains* (heEPODs) was included into the analysis as well. All selected integration sites should match the following criteria: (i) the influence on the expression of neighboring genes is minimized by avoiding integration downstream of a promoter or upstream of a terminator listed in RegulonDB [37], (ii) they can be assigned to the heEPODs described by Vora et al. [39], and (iii) they are at least 100 base pairs long to facilitate design of CRISPR/Cas targeting sequences as well as primer design for amplification of the homology arms for homologous recombination. A similar approach to identifying new integration loci, albeit with different search parameters, was described by Goormans et al. [34]. The homology arms of the donor DNA template were designed to be 500 bp in length, which results in higher integration efficiency compared to shorter variants [40, 41]. However, as our toolbox was designed to be used in both K-12 and B strains, 500 bp homology arms without single nucleotide polymorphisms had to be identified by comparing sequences of *E. coli* BL21(DE3) (GenBank accession no. CP001509.3) and the industrially relevant *E. coli* W3110 (GenBank accession no. AP009048.1). This led to the exclusion of several integration sites that met all previous search criteria. Finally, six integration loci were chosen for further experiments (Table S1), of which only one had been previously described in the literature [26, 34]. After cloning of the appropriate targeting sequences for CRISPR/Cas into the pgRNA plasmids, linear copies of the plasmids were amplified by PCR and fused with corresponding homology arms as well as the multiple cloning sites derived from either pET-28a(+) or pETDuet-1 to yield the pgRNA-ET or pgRNA-DUET vector series, respectively (Fig. 1).

### Evaluation of selected integration loci

The selected integration sites were evaluated using the fluorescent reporter protein mCherry. The *mCherry* gene was cloned into the pgRNA-ET plasmid series and subsequently integrated into the chromosomes of *E. coli* BL21(DE3) and *E. coli* W3110(T7) to compare possible differences between these representative B and K-12 strains. Although *E. coli* BL21(DE3) is considered to be hard-to-edit [42, 43], high integration efficiencies of up to 100% of all screened colonies (*n* = 20) were achieved (Table S1 & Figure S1). For reference purposes, *mCherry* was also integrated into the gene-disrupting *lacZ* locus which was previously described as a medium-to-low-expression locus [26, 34].

**Fig. 2:**
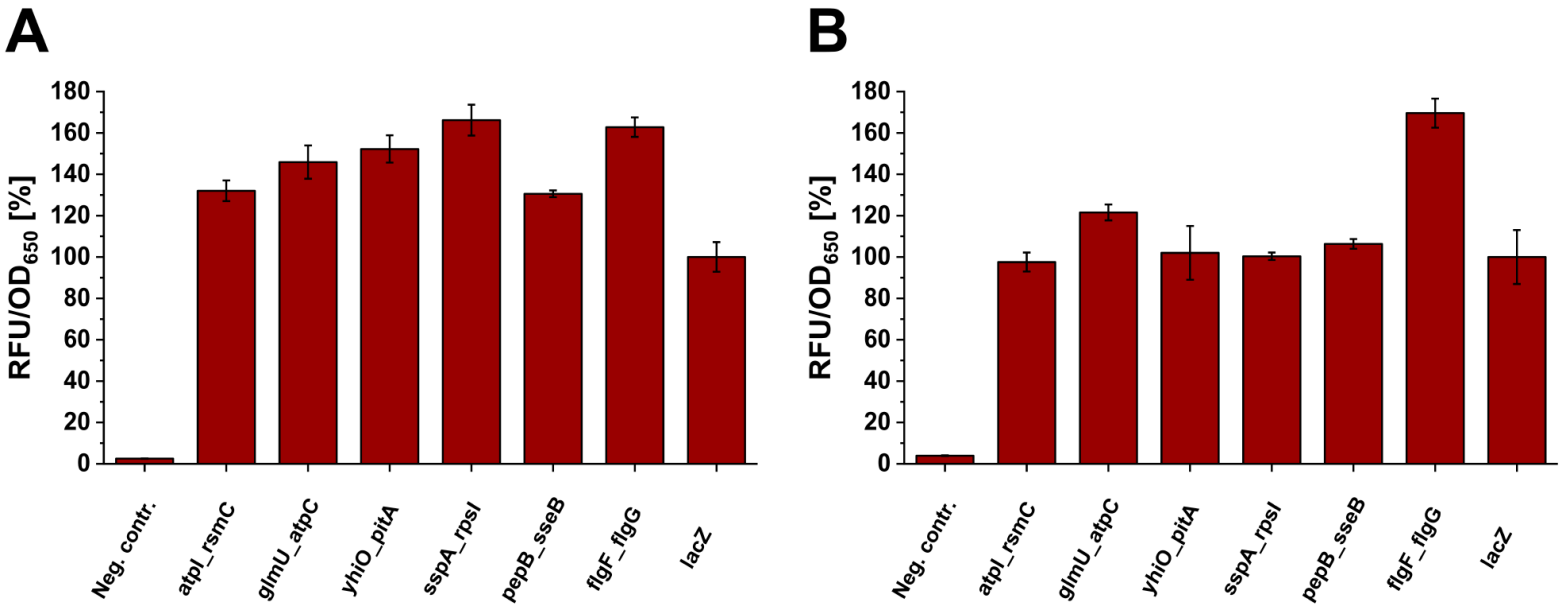
Relative fluorescence normalized to optical density (OD_650_) of cell cultures after chromosomal expression of *mCherry* integrated into different genomic loci in (A) *E. coli* BL21(DE3) and (B) *E. coli* W3110(T7). Strains harboring no copy of *mCherry* were used as negative controls to quantify intrinsic fluorescence of *E. coli*. Relative fluorescence was calculated in relation to *lacZ*. Means and standard deviations were calculated from three biological replicates

The expression of the chromosomally integrated *mCherry* gene showed no noticeable effect on cell growth (Figure S2). Compared to the *lacZ* reference locus, all *mCherry* integrations into the selected intergenic loci resulted in higher or at least similar fluorescence for both strains (Fig. 2).

In *E. coli* BL21(DE3), a 30–70% increase in fluorescence compared to *lacZ* was observed for all selected intergenic loci (*p* < 0.02). For *E. coli* W3110(T7), only integration into *flgF_flgG* (70% increase, *p* < 0.01) and *glmU_atpC* (20% increase, *p* = 0.04) loci resulted in higher expression compared to *lacZ*. It should be noted that after gene integration into the six intergenic loci, only low basal non-induced expression was observed, while after integration into *lacZ*, high fluorescence was observed without addition of isopropyl β-d-1-thiogalactopyranoside (IPTG) (Figure S3). A correlation between the fluorescence output and the distance from *oriC* - the above mentioned ‘gene dosage effect’ due to chromosome replication - could not be observed in our case. However, Bryant et al. reported that the gene dosage effect only accounted for 1.4-fold differences in expression [31]. Out of the six intergenic loci tested, *atpI_rsmG* had been described as one of the loci leading to the highest expression levels [26, 34]. Three of the newly characterized loci reached even higher fluorescence in *E. coli* BL21(DE3) (*p* < 0.05). It should be noted that unlike the gene-disrupting integration into the flagellar region 1 described by Juhas et al. [44], the integration between the genes *flgF* and *flgG* in *E. coli* W3110(T7) led to high expression combined with no loss of motility. Furthermore, plasmid-based expression using the pET-28a(+) vector showed that a single chromosomal copy reached up to 20% fluorescence compared to the vector which is reported to give about 40 gene copies per cell [45]. The *flgF_flgG* locus was chosen as the most promising integration site for further experiments due to the higher fluorescence observed in both strains and particularly in *E. coli* W3110(T7).

### Synthesis of coniferyl alcohol

First, the genes coding for the 4-coumarate-CoA ligase of *P. crispum* (*Pc*4CL) and the cinnamoyl-CoA reductase of *Z. mays* (*Zm*CCR) were cloned into a pETDuet vector for plasmid-based co-expression and preliminary tests for the synthesis of coniferyl alcohol **4.*** E. coli* W3110(T7), a K-12 derivative and industrially used strain, carrying this plasmid, was tested in two different media, terrific broth (TB) and lysogeny broth (LB), for the conversion of 5 mM ferulic acid **1** with growing cells. The substrate was added 2 h after induction. It was found that higher coniferyl alcohol **4** titers were reached in TB medium compared to LB medium 18 h after substrate addition (Figure S4). Thus, all following experiments were carried out using TB medium.

The same plasmid was then introduced into *E. coli* BL21(DE3) which is considered more suitable for heterologous expression due to its lack of Lon and OmpT proteases. Next, both heterologous genes were integrated into the *flgF_flgG* locus of both *E. coli* strains in a single step using the pgRNA-DUET vector. This allowed comparison of plasmid-based and plasmid-free expression as well as biotransformation in both strains.

**Fig. 3:**
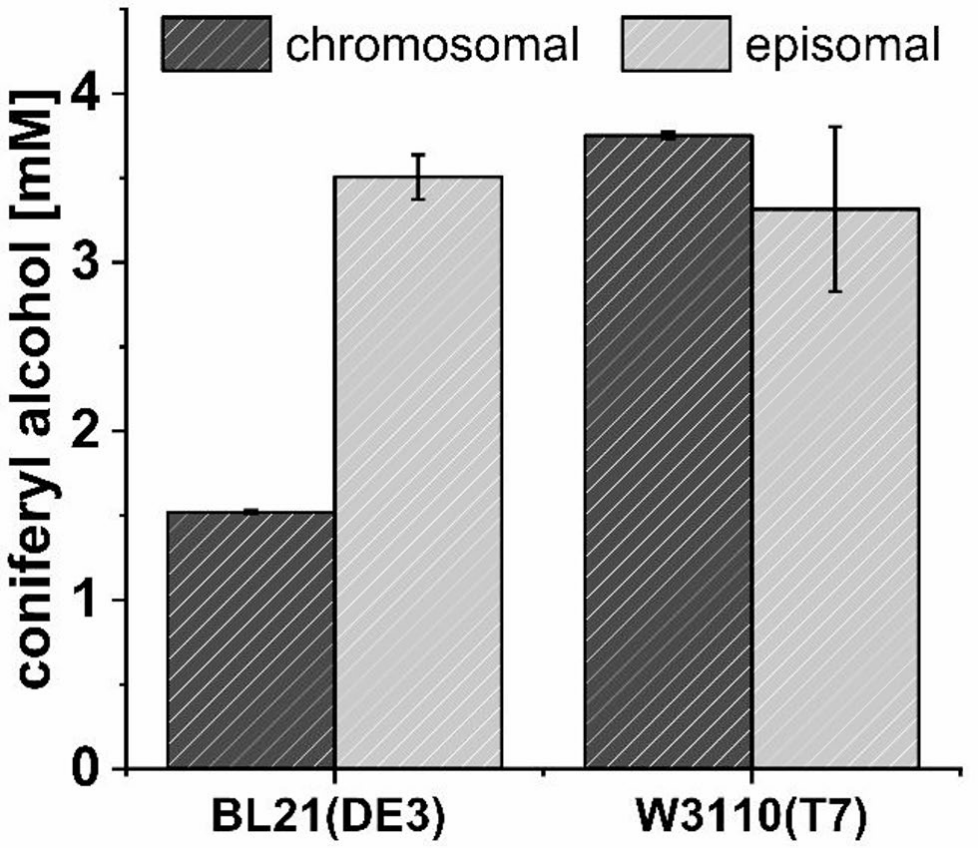
Coniferyl alcohol **4** synthesized by different *E. coli* strains 18 h after addition of 5 mM ferulic acid **1**. Expression of chromosomally integrated genes was compared to a pETDuet vector in *E. coli* BL21(DE3) and W3110(T7). Means and standard deviation were calculated from three biological replicates

Interestingly, the plasmid-based expression system performed equally well in both strains and yielded 3.5 ± 0.1 mM and 3.3 ± 0.5 mM coniferyl alcohol **4** (Fig. 3).

After chromosomal integration in BL21(DE3), however, only 1.52 ± 0.01 mM **4** was obtained. In general, lower expression levels and a therefore lower overall activity of the growing *E. coli* cells were expected due to the reduced copy number compared to the plasmid-based system. A striking exception for this was *E. coli* W3110(T7), which showed a coniferyl alcohol **4** titer comparable to the plasmid-based expression (3.75 ± 0.02 mM, 75% theoretical yield). The differences in product titers between plasmid-based and chromosomal expression systems can be mainly attributed to differences in heterologous expression of *ZmCCR* as visualized via Western Blot (Figure S5). Although *ZmCCR* appeared to be better expressed in *E. coli* BL21(DE3) compared to *E. coli* W3110(T7) when using plasmids, it was strongly affected by chromosomal integration, which reduced expression in this strain. In contrast, chromosomal integration in *E. coli* W3110(T7) did not result in lower protein concentration which may explain similar levels after plasmid-based and chromosomal expressions in this strain. In addition, minor differences in cell growth of the chromosomal expression strains were observed (*p* = 0.02): whereas *E. coli* W3110(T7) grew to an OD_600_ of 5.1 ± 0.2, *E. coli* BL21(DE3) only reached 4.3 ± 0.3 (Figure S6). Without the addition of the substrate, ferulic acid **1**, both strains reached significantly higher (*p* < 0.01) optical densities of 8.6 ± 0.1 and 7.8 ± 0.2, respectively. Since a concentration of 5 mM ferulic acid **1** does not affect the cell growth of *E. coli* as demonstrated by Yoon et al. [46], this difference may be due to either the metabolic burden caused by the consumption of cofactors during the reaction or the toxicity of the by-product coniferyl aldehyde **3**. Like many related phenolic aldehydes, **3** can be considered toxic to the cells and is therefore suspected to be reduced to the less toxic coniferyl alcohol **4** by multiple aldo-keto reductases or dehydrogenases in *E. coli* [18, 47]. In the literature, the combination of 4CL, CCR and heterologous cinnamyl alcohol dehydrogenases (CAD) has been used to produce 0.6 mM **4** from 1 mM **1** (60% yield) [48] and 1.82 mM **4** from 2.5 mM **4** (73% yield) [49] with plasmid-based whole-cell systems, clearly emphasizing the significance of our yield of 75% using single gene copies in the chromosome. However, an alternative plasmid-based approach using a carboxylic acid reductase instead of 4CL/CCR in combination with a heterologous aldo-keto reductase reached even 97% yield from 5 mM **1** [19].

**Fig. 4:**
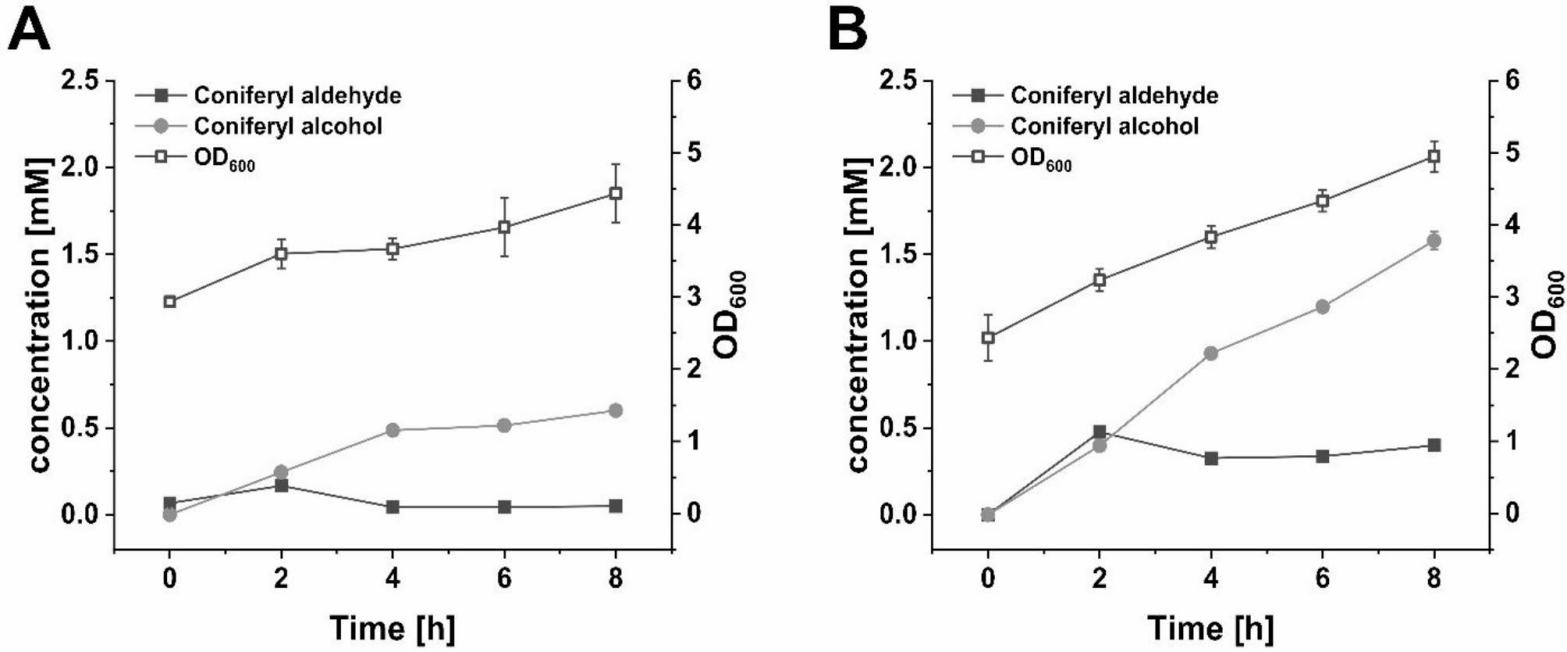
Time course of coniferyl aldehyde **3** and alcohol **4** formation as well as cell growth (OD_600_) during conversion of 5 mM ferulic acid **1** by two different plasmid-free whole-cell systems: (A) *E. coli* BL21(DE3) and (B) *E. coli* W3110(T7). Means and standard deviation were calculated from three biological replicates

To further investigate the differences between both strains after chromosomal integration and to gain insight into the metabolic flux, time course experiments were performed. Samples were taken every 2 h after substrate addition and the concentration of both the product coniferyl alcohol **4** and the intermediate coniferyl aldehyde **3** as well as the optical density were quantified (Fig. 4).

The higher productivity of *E. coli* W3110(T7) compared to *E. coli* BL21(DE3), as shown in Fig. 2, becomes already evident within the first few hours of conversion, despite no significant differences in cell growth. After 8 h, the coniferyl alcohol **4** concentration reached 0.60 ± 0.01 mM in *E. coli* BL21(DE3) and 1.58 ± 0.05 mM in *E. coli* W3110(T7). Coniferyl aldehyde **3** remained at constantly low levels and its highest concentrations of 0.17 ± 0.01 mM and 0.48 ± 0.02 mM, respectively, were reached only two hours after substrate addition. Interestingly, we noted an intense orange coloration and a characteristic odor of the cultures after substrate addition. The intensity of the orange color could be correlated with the detection of **3**, an orange substance, via LC/MS.

### Pinoresinol production

The *E. coli* W3110(T7) strain was chosen to extend the cascade starting from ferulic acid **1** to pinoresinol **5** due to its higher productivity for coniferyl alcohol synthesis compared to *E. coli* BL21(DE3). Based on our previous results [21], the laccase *Cg*L1 from *C. glutamicum* was selected as a promising candidate. Laccase-catalyzed oxidative coupling of two coniferyl alcohol **4** molecules proceeds via a radical mechanism. The phenol radical is stabilized by delocalization and can be represented by three different resonance forms (Figure S7). Thus, different coupling products are formed, of which pinoresinol **5** results from 8,8’-coupling and accounts for ~ 28% of all possible products in vitro [50]. Laccases contain four copper ions responsible for enzyme activity, and the reconstitution of the activity of *Cg*L1 in recombinant hosts like *E. coli* depends on copper availability. For this purpose, copper ions, for instance in form of CuSO_4_, are usually added at the time point of induction of laccase expression [51]. However, in the case of the reaction described here, there are a few aspects to consider. In fact, coniferyl alcohol **4** is not the only potential substrate of the laccase, because other phenolic compounds like ferulic acid **1** and coniferyl aldehyde **3** are confirmed or suspected substrates for homo- and cross-coupling reactions as well [51]. Indeed, during preliminary tests for laccase-mediated phenol coupling, cross-coupled dimers of coniferyl aldehyde **3** and coniferyl alcohol **4** were detected along with dimers of coniferyl alcohol **4**. The assignment of possible respective coupling products was achieved using in vitro reactions with FeCl_3_ as oxidizing agent (Figures S8-S10).

The equilibrium of the reduction of the aldehyde **3** to the alcohol **4** seemed to depend on the redox state of the cells. The addition of CuSO_4_ increased the oxidative stress [52], which in turn resulted in high aldehyde **3** titers and a higher amount of coniferyl aldehyde-alcohol cross-coupled products (data not shown). Unfortunately, one of these coupling products showed the same retention time in LC/MS as pinoresinol **5** and could not be resolved even by changing the analytical conditions (Figure S10). In our previous study, the addition of glucose or glycerol to the resting cells increased pinoresinol **5** formation [14]. As TB medium used in this study already contained glycerol, we tested additional glucose supplementation. The addition of 5 g/L glucose 0 h, 18 h and 42 h after substrate addition completely prevented aldehyde **3** accumulation and the subsequent formation of cross-coupling products (Figure S11). Apart from the NADPH-dependent reduction of **2** to **3** (Scheme 1), various endogenous NAD(P)H-dependent aldo-keto reductases and dehydrogenases like YqhD and YahK contribute to the subsequent reduction of **3** to **4** [18, 19, 53]. The addition of glucose strongly minimized the reoxidation of coniferyl alcohol **4** back to coniferyl aldehyde **3**, probably due to the change in the redox state in the cell and the increased availability of reduced nicotinamide cofactors [14]. In addition, changes in transcriptional regulation and expression of these endogenous enzymes might contribute to the observed effects of glucose addition.

Furthermore, the final product pinoresinol **5** also represents a substrate for laccases. In our previous in vitro studies, decrease of pinoresinol **5** was observed at a certain point of reaction dependent on the activity and the redox potential of the chosen laccase [21].

**Fig. 5:**
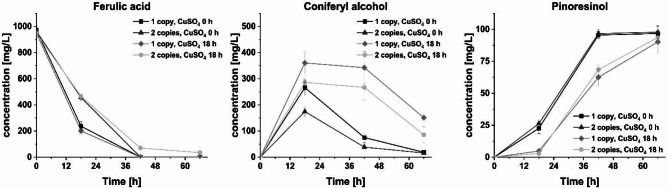
Time course of ferulic acid **1**, coniferyl alcohol **4** and pinoresinol **5** titers. The effect of delayed CuSO_4_ addition (at time of induction compared to 18 h after induction) and copy number of *cgl1* (one or two copies) were compared after addition of 970 mg/L (5 mM) ferulic acid **1**. Exemplary HPLC chromatograms are shown in Figure S11

For plasmid-free pinoresinol **5** synthesis, the *Cg*L1-coding gene was cloned into the pgRNA-ET vector series for subsequent chromosomal integration at two different sites. The first gene copy was integrated into *pepB_sseB* locus of *E. coli* W3110(T7). An additional gene copy was integrated into *atpI_rsmG* locus to investigate the effects of the gene copy number on pinoresinol **5** production. To determine whether the timing of CuSO_4_ addition provides advantages in the formation and preservation of pinoresinol **5**, addition at the time of induction (0 h) was compared to delayed addition after 18 h for both one and two integrated copies of *cgl1* (Fig. 5).

Interestingly, the strain harboring two laccase gene copies in the chromosome showed a slower ferulic acid **1** conversion and lower coniferyl alcohol **4** concentrations, but no influence on pinoresinol **5** formation was observed. Apparently, the integration of the second laccase gene copy affected the conversion of **1** to **4** negatively, which might be explained by altered expression levels of *Pc*4CL. Unfortunately, this hypothesis could not be confirmed via SDS-PAGE since the protein band on the gel did not appear prominent enough (Figure S12). However, the increased expression level of the laccase *Cg*L1 could be confirmed, but the titer of **5** did not increase further compared to a single gene copy. Pinoresinol **5** is a known substrate of *Cg*L1 and can therefore be degraded over time [21]. This might also explain why an equilibrium was reached between coniferyl alcohol **4** coupling and pinoresinol **5** degradation during the observation period: coniferyl alcohol **4** titers decreased but the concentration of **5** remained constant. In addition, insufficient incorporation of copper ions into the laccase protein has often been reported as a limiting factor upon laccase expression in recombinant *E. coli*, which may explain similar laccase activity regardless of gene copy number [51, 54, 55].

The time of CuSO_4_ addition clearly affected the laccase-catalyzed oxidative coupling: When CuSO_4_ was added at the time of induction, pinoresinol **5** could already be detected after 18 h and reached its maximum after 42 h. In cases where CuSO_4_ was added 18 h after induction, a comparable titer was only reached after 66 h. This difference was also reflected by the time course of the coniferyl alcohol **4** titers: When CuSO_4_ was added at the time of induction, lower titers of **4** were observed as it was already converted to its dimers by *Cg*L1. However, the gene expression is induced by IPTG and the same amount of proteins are therefore visible on the SDS-PAGE gel regardless of the time of CuSO_4_ addition (Figure S12).

Finally, a maximum concentration of ~ 100 mg/L (~ 270 µM) pinoresinol **5** was achieved using *E. coli* W3110(T7). Considering that 5 mM of substrate could yield a maximum of 2.5 mM dimeric coupling products, this corresponds to a theoretical yield of 10.8% with respect to the amount of ferulic acid **1** used in the reaction. Since the maximum titer of coniferyl alcohol **4** from 5 mM **1** was only 3.75 mM (Fig. 3), the yield of pinoresinol **5** with respect to coniferyl alcohol **4** was 14.4%. Our previously reported approach using recombinant *E. coli* resting cells and the endogenous *E. coli* multicopper oxidase CueO for coniferyl alcohol **4** coupling reached 240 µM pinoresinol **5** starting from 5 mM substrate (9.6% yield) on 0.5 mL scale [22]. In this case, resting cells were applied at higher cell densities. With purified enzymes, even 4.4 mM pinoresinol **5** starting from 10 mM eugenol could be achieved [21]. The higher yield of 17.6% might be attributed to the use of a biphasic in vitro system that might have prevented degradation of **5**. Nevertheless, it should be noted that an *in vitro* setup with isolated/purified enzymes as well as the use of resting cells require significantly more work compared to the growing cell approach presented here.

Since upscaling attempts with 10 mM ferulic acid **1** led to lower substrate conversion (data not shown), for preparative synthesis of pinoresinol **5**, 5 mM ferulic acid **1** was converted using the strain harboring one *cgl1* copy at 400 mL scale. CuSO_4_ was added at the time of induction and the products were extracted after 66 h of conversion. After flash chromatography using both normal and reversed phase silica, 43 mg of pinoresinol (> 98% purity quantified via HPLC, Figure S13) were isolated. Product identity was verified by LC/MS (ESI(-) m/z 357 [M-H]^−^) and nuclear magnetic resonance (NMR) spectroscopy. The results of ^1^H-NMR (Figure S14) and ^13^C-NMR (Figure S15) were consistent with published data [23].

## Conclusions

In this study, the evaluation of six intergenic integration loci using the fluorescent reporter mCherry allowed the selection of suitable loci for plasmid-free heterologous expression of plant and bacterial genes and subsequent biotransformation in growing *E. coli* cells. A versatile plasmid toolbox for the transfer of genes from pET vectors into these intergenic loci in both *E. coli* B and K-12 strains was established and evaluated. The application of this toolbox was demonstrated by integrating the genes encoding enzymes for the conversion of ferulic acid **1** to pinoresinol **5** in *E. coli* BL21(DE3) and W3110(T7). In future studies, this toolbox could also be used to extend the developed biosynthetic path and introduce it into a tyrosine-overproducing *E. coli* strain that use the cheaper substrate glucose. The synthesis of coniferyl alcohol **4** revealed substantial differences between the two strains tested. In *E. coli* W3110(T7), the chromosomal expression system enabled the same coniferyl alcohol **4** titer (3.75 mM) as the corresponding plasmid-based expression system. The reduction of coniferyl aldehyde **3** to alcohol **4** was catalyzed by endogenous enzymes of the host. Here, the supplementation of glucose to the medium shifted the equilibrium towards the formation of **4**. Finally, a maximal concentration of 100 mg/L pinoresinol **5** was reached. This study not only provided a nature-inspired route for the synthesis of pinoresinol **5**, but also helped to facilitate the progress towards plasmid-free enzymatic cascades in *E. coli*.

## Methods

### Strains and plasmids

*E. coli* DH5α was used for cloning and for plasmid propagation. *E. coli* BL21(DE3) and W3110(T7) [56] were used for expression, genome engineering and biotransformation. A list of all strains used and created in this study can be found in the supporting information (Table S2). Plasmids used for expression, as template for gene amplification or chromosomal integration are listed in Table S3.

### Construction of pET-28a(+)_mCherry and pETDuet_ZmCCR_Pc4CL

The gene *mCherry* was amplified from pSEVA227R and cloned into the pET-28a(+) empty vector (Novagen) using FastDigest NdeI and EcoRI restriction enzymes and T4 DNA Ligase (Thermo Fisher Scientific). Digestion and subsequent ligation of the digested genes and vectors was carried out according to the manufacturer’s protocols. A list of all primers used in this study can be found in the supporting information (Table S4).

The genes encoding the 4-coumarate-CoA ligase from *P. crispum* (*Pc*4CL) and the cinnamoyl-CoA reductase from *Z. mays* (*Zm*CCR) were amplified from pETDuet-TAL-4CL and pRSFDuet-CCR-CAD [16], respectively, and cloned into the pETDuet-1 empty vector (Novagen). Restriction digestion of the amplified genes and vector was performed using FastDigest NdeI and XhoI for cloning of *Pc4CL* and FastDigest EcoRI and NotI for cloning of *ZmCCR* (Thermo Fisher Scientific).

Chemically competent *E. coli* DH5α cells were transformed with the respective ligation product. The constructed plasmids were isolated from 4 ml of overnight cultures using the ZR Plasmid Miniprep – Classic Kit from ZymoResearch. Sequence integrity was verified by Sanger sequencing (Eurofins Genomics).

### Construction of the pgRNA-ET and pgRNA-DUET vector series and chromosomal integration

The basic steps for CRISPR/Cas-assisted chromosomal integration were carried out as described before for linear donor DNA [56]. In brief summary, the design and evaluation of locus-specific sgRNAs was performed using the CHOPCHOP v3 web tool [57] (for details see Table S5). The N_20_ targeting sequence of the plasmid pgRNA was substituted for each of the chosen loci by PCR. The primers contained the locus-specific N_20_ as well as a BcuI (SpeI) restriction site for subsequent circularization of the linear PCR products (Table S4). Digestion with FastDigest BcuI and DpnI (Thermo Scientific) to remove the methylated template DNA was followed by a self-circularization ligation step as described by Seo et al. [43]. Successful substitution was verified by Sanger sequencing (Eurofins Genomics).

For λ-Red-mediated homologous recombination, homology arms of ~ 500 bp suitable for both *E. coli* K-12 and B strains were designed using the genome sequences of *E. coli* W3110 (GenBank accession no. AP009048.1) and *E. coli* BL21(DE3) (GenBank accession no. CP001509.3). These sequences were amplified by PCR from boiled cells of *E. coli* BL21(DE3). Linear double-stranded donor DNA (dsDonorDNA) for chromosomal integration into the *lacZ* reference locus was generated by fusion PCR of homology arms and *mCherry* as described before [56].

For construction of the pgRNA-ET and pgRNA-DUET vector series, the different pgRNA plasmids containing the locus-specific targeting sequences were linearized by PCR. Corresponding amplified homology arms and expression cassettes from pET-28a(+) (for construction of the pgRNA-ET series) or pETDuet-1 (for construction of the pgRNA-DUET series) were recombined as described by Gibson et al. [58]. In some cases, fusion PCR of the homology arms and the expression cassettes prior to Gibson assembly was used to improve the cloning efficiency. An illustration of the entire cloning procedure can be found in Figure S16.

The expression cassette of pgRNA-ET consists of the T7 promoter, a ribosome binding site, one multiple cloning site containing different optional tags, and the T7 terminator. In contrast, the expression cassette of pgRNA-DUET contains two multiple cloning sites, each with an upstream T7 promoter and ribosome binding site, allowing for two transcripts: a monocistronic transcript of the gene located in multiple cloning site no. 2, and a bicistronic transcript of genes of both multiple cloning sites.

For chromosomal integration of heterologous genes using the created toolbox, the genes to be integrated were amplified from the respective pET vectors by standard PCR using Phusion High-Fidelity DNA Polymerase (Thermo Scientific) according to the manufacturer’s protocol. After purification by agarose electrophoresis, the amplicons as well as pgRNAET vectors were digested using FastDigest NdeI/XhoI for *mCherry* and FastDigest BamHI/NcoI for the laccase gene *cgl1* as described in the manufacturer’s manual. Vectors and inserts were ligated using T4 DNA Ligase (Thermo Scientific) and subsequently used for transformation of chemically competent *E. coli* DH5α. Plasmids of selected colonies were isolated using the ZR Plasmid Miniprep – Classic kit (Zymo Research) and sequenced using standard T7/T7term primers (Eurofins Genomics).

The laboratory steps for chromosomal integration were carried out as previously described [56]. In brief, electrocompetent cells harboring pEcCas for expression of cas9 and λ-Red genes were freshly prepared. For this purpose, 5 ml 2xYT medium (containing 30 µg/ml kanamycin and 20 mM L-arabinose) was inoculated with 100 µl of an overnight culture and incubated at 37 °C with shaking at 180 revolutions per minute (rpm) for 2 h. Cells were harvested by centrifugation and washed twice with 1 ml ice-cold 10%(v/v) glycerol. After the cell pellet was resuspended in 100 µl 10%(v/v) glycerol, 100 ng of the circular pgRNA-ET or pgRNA-DUET plasmids were added which provided both the sgRNA and the necessary repair template for homologous recombination. After electroporation (2.5 kV, MicroPulser™ electroporator from BioRad), the cells were immediately transferred to 1 ml of SOC medium and incubated at 37 °C, 180 rpm for one hour. Finally, the cells were plated out on LB agar plates (30 µg/ml kanamycin, 100 µg/ml ampicillin) and incubated at 37 °C overnight. Successful integration was verified by colony PCR using the primers listed in Table S4. Primers were designed to amplify both with and without successful integration, so that integration could be identified by the length of the amplicons (as seen in Figure S1). Plasmid curing was performed iteratively in 5 ml LB medium with the addition of 10 mM L-rhamnose for removing pgRNA-ET or pgRNA-DUET and 5%(w/v) sucrose for pEcCas, respectively.

### Chromosomal expression and quantification of mCherry

After chromosomal integration into all loci in *E. coli* BL21(DE3) and *E. coli* W3110(T7), the fluorescent reporter *mCherry* was expressed in 50 ml LB medium, which was inoculated with 1 ml of an overnight culture. The cultures were grown at 37 °C, 180 rpm until an OD_600_ of 0.6–0.7 was reached. After addition of 0.1 mM IPTG for induction, the expression was performed at 30 °C, 180 rpm for 20 h. The optical density was now quantified at 650 nm to avoid interference with the fluorescence of mCherry [59]. Fluorescence measurements of 200 µl culture aliquots were performed using a Tecan Infinite M200 Pro microplate reader (excitation at 570 nm, emission at 625 nm).

### Heterologous expression and biotransformation of ferulic acid

The genes encoding *Pc*4CL and *Zm*CCR were integrated into the *flgF_flgG* locus of the chromosomes of *E. coli* strains BL21(DE3) and W3110(T7). To compare chromosomal and plasmid-based expression, both strains (without integrated genes) were also transformed with pETDuet_ZmCCR_Pc4CL. The laccase gene *cgl1* was subsequently integrated into the loci *pepB_sseB* and *atpI_rsmG* of *E. coli* W3110(T7) already harboring copies of *Pc4CL* and *ZmCCR* in the *flgF_flgG* locus.

For biotransformation, the respective *E. coli* cells were grown in TB or LB medium. Cultures were inoculated with 2%(v/v) of an overnight culture in either 50 ml for analytical reactions or 400 ml scale for the preparative reaction (cf. 4.7) and incubated at 37 °C, 180 rpm. For plasmid-based expression, 30 µg/ml kanamycin was used to maintain the pETDuet plasmid in the cell. At an OD_600_ of 0.6 to 0.8, transcription was induced by addition of 0.1 mM IPTG and the cells were subsequently incubated at 30 °C, 140 rpm. After 2 h, 5 mM ferulic acid **1** was added using a stock solution of 250 mM in dimethyl sulfoxide (DMSO), resulting in a final concentration of 2%(v/v) DMSO. To prevent aldehyde **3** formation, 5 g/L glucose was supplemented at time of substrate addition, after 18 h and after 42 h. For copper loading of the laccases, 3 mM CuSO_4_ was added either at time of induction or 18 h after substrate addition. For quantification of substrate and products as well as SDS-PAGE analysis and Western Blot, 500 µl samples were taken at different time points.

For product analysis, 125 µl of culture medium was diluted with 115 µl water. Cinnamic acid (62.5 mM dissolved in DMSO) was added as an internal standard (10 µl, 2.5 mM final concentration). Extraction was performed twice using 500 µl ethyl acetate each. The combined organic phase (2 × 400 µl) was evaporated and the residue was dissolved in 100 µl methanol. Product analysis was performed via HPLC and LC/MS analysis using a gradient of water with 0.1%(v/v) formic acid (A) and methanol (B) at a flow rate of 0.5 ml/min (LCMS-2020, Shimadzu; Chromolith Performance RP-18e 100 –4.6 mm column and Chromolith RP-18e 10 –4.6 mm guard cartridge, Merck Millipore). Details of the HPLC gradient settings are provided in Table S6. The LCMS-2020 was set to scan mode to detect both positive and negative ions using the dual ion source (m/z 159–1000, scan speed 3750 u/s, event time 0.25 s). Products were identified by comparison of retention time and mass to charge ratio (m/z) with commercial standards. Substrate and products were quantified based on their UV absorption using a photodiode array detector (SPD-M20A, Shimadzu) at different wavelengths (ferulic acid **1** 325 nm, coniferyl aldehyde **3** 340 nm, coniferyl alcohol **4** 260 nm, pinoresinol **5** 280 nm). Linear internal calibration curves of all three compounds were used to calculate the substrate and product titers from peak areas at the indicated wavelengths for each sample.

For SDS-PAGE analysis, aliquots of the cell suspension equivalent to an OD_600_ of 0.25 were centrifuged. The cell pellets were resuspended in 50 µl 1 x SDS-PAGE loading buffer and loaded onto 12.5% polyacrylamide gels [60]. For Western blot analysis, proteins were blotted onto a nitrocellulose membrane which was subsequently washed twice with TBS (20 mM Tris-HCl, 150 mM NaCl, pH 7.6) and blocked with 3%(w/v) bovine serum albumin (BSA) in TBS. After washing with TBST (0.1%(v/v) Tween 20 in TBS) and TBS, the membrane was incubated with a 1:1000 dilution of the primary antibody (mouse 6x-His tag monoclonal antibody HIS.H8 from Thermo Fisher Scientific) in TBS with 3%(w/v) BSA for one hour. Prior to the incubation with the secondary antibody (Peroxidase AffiniPure™ Goat Anti-Mouse IgG (Jackson ImmunoResearch), 1:10,000 diluted in TBS with 10%(w/v) milk powder), the membrane was washed twice with TBST and once with TBS. Finally, the membrane was washed thoroughly with TBS and the color reaction was initiated by the addition of 3,3’,5,5’-tetramethylbenzidine (TMB) Liquid Substrate System (Sigma Aldrich).

### Oxidative coupling using FeCl_3_

For identification of cross-coupling products of coniferyl aldehyde **3** and alcohol **4**, 10 µl of 5 mM solutions in DMSO for homo-coupling or 5 µl each for cross-coupling were diluted in 230 µl ddH_2_O. Addition of 10 µl 5 mM FeCl_3_ (equimolar amount) at room temperature led to the formation of coupling products within minutes. The mixture was extracted twice with 500 µl ethyl acetate. The combined organic phases were evaporated and the residue was dissolved in 50 µl methanol for LC/MS analysis.

### Isolation of pinoresinol

For product isolation and purification, two 400 ml cultures for conversion of 5 mM ferulic acid **1** were incubated for 66 h (with glucose addition 0 h, 18 h, and 42 h after substrate addition). The reaction was monitored as described above. The cells were harvested by centrifugation and the supernatant was used for product extraction in a separation funnel. The aqueous solution was extracted with 2 × 400 ml ethyl acetate. The combined organic phases were dried with MgSO_4_, filtered and evaporated under reduced pressure. The residue was subjected to flash chromatography (Silica 60 M 0.04–0.063 mm, Macherey-Nagel) using a mixture of ethyl acetate (70%(v/v)) and n-heptane (30%(v/v)) as eluent. The addition of 2%(v/v) triethylamine to the eluent improved separation. Progress of flash chromatography was monitored using thin layer chromatography. Fractions containing a high amount of pinoresinol **5** were combined and analyzed via LC/MS. Since purification via normal phase was not sufficient, reversed-phase flash chromatography (C_18_-Reversed-Phase Silica, Sigma Aldrich) was performed. The mobile phase consisted of 50%(v/v) methanol in water. Combined fractions were first evaporated under reduced pressure to remove methanol. The remaining aqueous phase was freeze-dried. The purity of the isolated compound was analyzed via HPLC and the structure was confirmed by ^1^H- and ^13^C-NMR (Bruker Avance III – 300).

## Electronic supplementary material

Below is the link to the electronic supplementary material.

Supplementary Material 1

## Data Availability

No datasets were generated or analysed during the current study.
