# Supplementary material for: Plasmid-free production of the plant lignan pinoresinol in growing Escherichia coli cells
Source: Microb Cell Fact. 2024 Oct 23;23:289. doi: 10.1186/s12934-024-02562-3 (PMC11515702; doi:10.1186/s12934-024-02562-3)
Supplement: Supplementary file 1 — Supplementary Material 1 [file 12934_2024_2562_MOESM1_ESM.pdf]

Supporting information

Plasmid-free production of the plant lignan pinoresinol in growing *Escherichia coli* cells

U. Joost Luelf[1], Alexander Wassing[1], Lisa M. Böhmer[1], Vlada B. Urlacher[1]*

[1]Institute of Biochemistry, Heinrich Heine University Düsseldorf, 40225 Düsseldorf, Germany

*Corresponding Author

Email: vlada.urlacher@uni-duesseldorf.de

ORCID: Vlada B. Urlacher 0000-0003-1312-4574

Running title: Plasmid-free pinoresinol synthesis

Supporting Tables

Table S1. Efficiency of *mCherry* integration in *E. coli* BL21(DE3) using the pgRNA-ET vector series.

Locus	Integration efficiency
atpI_rsmG	15/20
glmU_atpC	16/20
yhiO_pitA	18/20
sspA_rpsI	19/20
pepB_sseB	19/20
flgF_flgG	20/20

Table S2. List of strains used in this study

Strain	Purpose	Source
E. coli DH5α	cloning procedures	Clontech
E. coli BL21(DE3)	expression and genome engineering	Novagen
E. coli W3110(T7)	expression and genome engineering	(1)
E. coli BL21(DE3) mCherry (atpI_rsmG)	expression	this study
E. coli BL21(DE3) mCherry (glmU_atpC)	expression	this study
E. coli BL21(DE3) mCherry (yhiO_pitA)	expression	this study
E. coli BL21(DE3) mCherry (sspA_rpsI)	expression	this study
E. coli BL21(DE3) mCherry (pepB_sseB)	expression	this study
E. coli BL21(DE3) mCherry (flgF_flgG)	expression	this study
E. coli BL21(DE3) mCherry (lacZ)	expression	this study
E. coli W3110(T7) mCherry (atpI_rsmG)	expression	this study
E. coli W3110(T7) mCherry (glmU_atpC)	expression	this study
E. coli W3110(T7) mCherry (yhiO_pitA)	expression	this study
E. coli W3110(T7) mCherry (sspA_rpsI)	expression	this study
E. coli W3110(T7) mCherry (pepB_sseB)	expression	this study
E. coli W3110(T7) mCherry (flgF_flgG)	expression	this study
E. coli W3110(T7) mCherry (lacZ)	expression	this study
E. coli BL21(DE3) ZmCCR_Pc4CL (flgF_flgG)	expression and biotransformation	this study
E. coli W3110(T7) ZmCCR_Pc4CL (flgF_flgG)	expression and biotransformation	this study
E. coli W3110(T7) ZmCCR_Pc4CL (flgF_flgG) Cgl1 (pepB_sseB)	expression and biotransformation	this study
E. coli W3110(T7) ZmCCR_Pc4CL (flgF_flgG) 2xCgl1 (pepB_sseB, atpI_rsmG)	expression and biotransformation	this study

Table S3. List of plasmids used in this study.

Plasmid	Purpose	Source
pEcCas	expression of cas9 and λ-Red genes	(2)
pgRNA-bacteria	transcription of sgRNA;<br>template for N_{20} exchange	(3)
pgRNA_001	targets *atpI_rsmG* locus	(1)
pgRNA_002	targets *glmU_atpC* locus	this study
pgRNA_003	targets *yhiO_pitA* locus	this study
pgRNA_004	targets *sspA_rpsI* locus	this study
pgRNA_005	targets *pepB_sseB* locus	this study
pgRNA_006	targets *flgF_flgG* locus	this study
pgRNA_007	targets *lacZ* locus	this study
pgRNA-ET_001	for integration in *atpI_rsmG* locus	this study
pgRNA-ET_002	for integration in *glmU_atpC* locus	this study
pgRNA-ET_003	for integration in *yhiO_pitA* locus	this study
pgRNA-ET_004	for integration in *sspA_rpsI* locus	this study
pgRNA-ET_005	for integration in *pepB_sseB* locus	this study
pgRNA-ET_006	for integration in *flgF_flgG* locus	this study
pSEVA227R	template for amplification of *mCherry*	SEVA
pET-28a(+) mCherry	template for cloning of *mCherry*	this study
pgRNA-ET_001_mCherry	for integration of *mCherry* in *atpI_rsmG* locus	this study
pgRNA-ET_002_mCherry	for integration of *mCherry* in *glmU_atpC* locus	this study
pgRNA-ET_003_mCherry	for integration of *mCherry* in *yhiO_pitA* locus	this study
pgRNA-ET_004_mCherry	for integration of *mCherry* in *sspA_rpsI* locus	this study
pgRNA-ET_005_mCherry	for integration of *mCherry* in *pepB_sseB* locus	this study
pgRNA-ET_006_mCherry	for integration of *mCherry* in *flgF_flgG* locus	this study
pgRNA-Duet_006	for integration of *mCherry* in *flgF_flgG* locus	this study
pRSFDuet_CCR_CAD	template for amplification of *ZmCCR*	(4)
pETDuet-TAL-4CL	template for amplification of *Pc4CL*	(4)
pETDuet_ZmCCR_Pc4CL	expression	this study
pgRNA-Duet_006_ZmCCR_Pc4CL	for integration of *ZmCCR* & *Pc4CL* in *flgF_flgG* locus	this study
pET-16b_cgl1	template for amplification of *cgl1*	(5)
pgRNA-ET_001_cgl1	for integration of cgl1 in *atpI_rsmG* locus	this study
pgRNA-ET_005_cgl1	for integration of cgl1 in *pepB_sseB* locus	this study

Table S4. Primer sequences.

Name	Integration Locus	Sequence (5‘ to 3‘)	Reference
Primers for amplification & cloning of mCherry into pET-28a(+) (Restriction sites are underlined)			
mCherry fw	-	ATATATCATATGATGGTGAGCAAGGGCGAGGAG	-
mCherry rev	-	ATATATGAATTCTTACTTGTACAGCTCGTCC	-
Primers for cloning of *mCherry* & *cgl1* into pgRNA-ET plasmids			
pET-28a(+) fw	-	TATAGGCGCCAGCAACC	-
pET-28a(+) rev	-	TTAATGCGCCGCTACAGG	-
cgl1 fw	-	CACCATGGGTCCCTCCCTTCGCCC	-
cgl1 rev	-	CGGGATCCTCCTTACTCGTAGCGAAGCGAG	-
Primers for amplification & cloning of *ZmCCR* and *Pc4CL* into pETDuet (Restriction sites are underlined)			
ZmCCR fw	-	ATATATGAATTCGATGACGGTGGTTGATGCG	-
ZmCCR rev	-	ATATATGCGGCCGCTTAGGCGCGAATTGCAATG	-
Pc4CL fw	-	ATATATCATATGGGAGATTGTGTAGCACC	-
Pc4CL rev	-	ATATATCTCGAGTTATTTGGGAAGATCACCGG	-
gRNA Primer (Restriction sites are underlined. N20 targeting sequences are shown in italic.)			
gRNA rev	-	ATATATACTAGTATTATACCTAGGACTGAGCTAG	general design (6)
gRNA_XXX fw (general primer design)	-	ATATATACTAGT-N_{20}-GTTTTAGAGCTAGAAATAGC	general design (6)
gRNA_001 fw	atpI_rsmG	ATATATACTAGT*TTATTAAAAATGTCAATGGG*GTTTTAGAGCTAGAAATAGC	(1)
gRNA_002 fw	glmU_atpC	ATATATACTAGT*TATGTGAACGCTATTCAGGA*GTTTTAGAGCTAGAAATAGC	-
gRNA_003 fw	yhiO_pitA	ATATATACTAGT*ACTATGTCACAATCTGAAGC*GTTTTAGAGCTAGAAATAGC	-
gRNA_004 fw	sspA_rpsI	ATATATACTAGT*TGTTTTACCTGTCTGTCAGG*GTTTTAGAGCTAGAAATAGC	-
gRNA_005 fw	pepB_sseB	ATATATACTAGT*ACAAATTGCGAATCCCTTGT*GTTTTAGAGCTAGAAATAGC	-
gRNA_006 fw	flgF_flgG	ATATATACTAGT*TTTTGCGAGCACTTGTAGGC*GTTTTAGAGCTAGAAATAGC	-
gRNA_007 fw	lacZ	ATATATACTAGT*ACTATGTCACAATCTGAAGC*GTTTTAGAGCTAGAAATAGC	-
pgRNA seq		GCCACCTGACGTCTAAG	(1)
Primers for cloning of pgRNA-ET and pgRNA-DUET			
pET fw + Overlap HA1		GCTAAGAACCATCATTGGCTGTATAGGCGCCAGCAACC	-
pET rev + Overlap HA2		GCTAACCCTGTTCGATCATTAATGCGCCGCTACAGG	-
HA1 fw	atpI_rsmG	GCCACCACCAGTAACAC	-
HA1 rev		TGATCGAACAGGGTTAGC	-
HA2 fw		CAGCCAATGATGGTTCTTAGC	-

Name	Integration Locus	Sequence (5‘ to 3‘)	Reference
HA2 rev		CGTCAGGTGCAACATGAG	-
pgRNA fw		GTGTTACTGGTGGTGGCTTCAAAAAAAGCACCGACTC	-
pgRNA rev		CTCATGTTGCACCTGACGGGGATAACGCAGGAAAGAAC	-
pET fw + Overlap HA1	glmU_atpC	GTGTGACCCGTCCTGAATATAGGCGCCAGCAACC	-
pET rev + Overlap HA2		CGTCAGGTGGATGTTTTTGTTAATGCGCCGCTACAGG	-
HA1 fw		GAGGGTGATATGGCAATGAC	-
HA1 rev		TTCAGGACGGGTCACAC	-
HA2 fw		CAAAAACATCCACCTGACGC	-
HA2 rev		CATCGGCAAAGAAAGGTG	-
pgRNA fw		GTCATTGCCATATCACCCTCTTCAAAAAAAGCACCGACTC	-
pgRNA rev		CACCTTTCTTTGCCGATGGGGATAACGCAGGAAAGAAC	-
pET fw + Overlap HA1	yhiO_pitA	CCAGTAAACTGACCCGCTTAATGCGCCGCTACAGG	-
pET rev + Overlap HA2		CTGTAATCGTACGCACCATATAGGCGCCAGCAACC	-
HA1 fw		AAGCGGTTTCTCCAGGAC	-
HA1 rev		GCGGGTCAGTTTACTGG	-
HA2 fw		TGGTGCGTACGATTACAG	-
HA2 rev		CGCCGCATTATGCTGTG	-
pgRNA fw		GTCCTGGAGAAACCGCTTTTCAAAAAAAGCACCGACTC	-
pgRNA rev		CACAGCATAATGCGGCGGGGATAACGCAGGAAAGAAC	-
pET fw + Overlap HA1	sspA_rpsI	CCCTTATTGGCGATGTGGTTTTAATGCGCCGCTACAGG	-
pET rev + Overlap HA2		GAATCAGCGTAAAAACTGGAATATAGGCGCCAGCAACC	-
HA1 fw		ATGATGCGAGATTCCCACAG	-
HA1 rev		AACCACATCGCCAATAAGGG	-
HA2 fw		TTCCAGTTTTTACGCTGATTC	-
HA2 rev		CAATACTACGGCACTGGTC	-
pgRNA fw		CTGTGGGAATCTCGCATCATTTCAAAAAAAGCACCGACTC	-
pgRNA rev		GACCAGTGCCGTAGTATTGGGGATAACGCAGGAAAGAAC	-
pET fw + Overlap HA1	pepB_sseB	CCTGATGCGCTACGCTTATTAATGCGCCGCTACAGG	-
pET rev + Overlap HA2		CTAATATGCCGGATGCGGCTATAGGCGCCAGCAACC	-
HA1 fw		ACCGCCTTCCAGGATTTC	-
HA1 rev		TAAGCGTAGCGCATCAGG	-
HA2 fw		GCCGCATCCGGCATATTAG	-
HA2 rev		AGCTGGGCGATATCATCACC	-
pgRNA fw		GGTGATGATATCGCCCAGCTTTCAAAAAAAGCACCGACTC	-
pgRNA rev		GAAATCCTGGAAGGCGGTGGGATAACGCAGGAAAGAAC	-
pET fw + Overlap HA1	flgF_flgG	CTGTAGTGGATTAGTGACAAAGTTAATGCGCCGCTACAGG	-
pET rev + Overlap HA2		GTATAAGTTGCCCGATGCGTATAGGCGCCAGCAACC	-

Name	Integration Locus	Sequence (5‘ to 3‘)	Reference
HA1 fw		GCGTAATGGCAGCATTC	-
HA1 rev		CGCATCGGGCAACTTATAC	-
HA2 fw		CTTTGTCACTAATCCACTACAG	-
HA2 rev		TCACGACCGATGGTGATAC	-
pgRNA fw		GTATCACCATCGGTCGTGATTCAAAAAAAGCACCGACTC	-
pgRNA rev		GAATGCTGCCATTACGCGGGATAACGCAGGAAAGAAC	-
Primers for synthesis of linear dsDonorDNA (*lacZ* locus)			
pET fw + Overlap HA1	lacZ	GTCTTCATCCACGCGTTAATGCGCCGCTACAGG	-
pET rev + Overlap HA2		CCATGTTGCCACTCGTATAGGCGCCAGCAACC	-
HA1 fw		ACTGTGAGCCAGAGTTG	-
HA1 rev		CGCGTGGATGAAGAC	-
HA2 fw		CGAGTGGCAACATGG	-
HA2 rev		TGGCGTAATAGCGAAG	-
Primers for verification of integration by colony PCR			
atpI_rsmG fw	atpI_rsmG	TCAGCGGCAAGAATACC	-
atpI_rsmG rev		TCCTGAAGCCCATTTCAC	-
glmU_atpC fw	glmU_atpC	GGAAGGCGAATACGATCAC	-
glmU_atpC rev		GCGGTTAAAGGCATGTTG	-
yhiO_pitA fw	yhiO_pitA	TCAATCCGCCTTGCTTAC	-
yhiO_pitA rev		ACCATTAACGCGCTCAAC	-
sspA_rpsI fw	sspA_rpsI	GCGGGTCATATAGCCTTTC	-
sspA_rpsI rev		GGAAGGCGAATACGATCAC	-
pepB_sseB fw	pepB_sseB	GCTTCACCGGCTTAATGG	-
pepB_sseB rev		CGGCTACAGCATCAAACAG	-
flgF_flgG fw	flgF_flgG	GTGGAAGGGCTTTCTCTG	-
flgF_flgG rev		TTCGCCAATGCTCTCC	-
lacZ fw fw	lacZ	TATAGGCGCCAGCAACC	-
lacZ rev rev		ACTGTGAGCCAGAGTTG	-

Table S5: Positions of cut sites in the genome of *E. coli* BL21(DE3) (GenBank accession no. CP001509.3) and *E. coli* W3110 (GenBank accession no. AP009048.1).

**Locus**	**Position of target site in *E. coli* BL21(DE3)**	**Position of target site in *E. coli* W3110**
atpI_rsmG	3,811,627	3,713,854
glmU_atpC	3,804,242	3,721,239
yhiO_pitA	3,501,563	4,001,236
sspA_rpsI	3,238,863	3,377,555
pepB_sseB	2,520,924	2,653,655
flgF_flgG	1,137,219	1,136,229

Table S6: HPLC gradient settings. Mobile phase consists of water with 0.1%(v/v) formic acid (A) and methanol (B). Flow rate was set to 0.5 ml/min and column oven temperature was set to 30 °C.

**Time [min]**	**concentration of methanol (B) [%]**
0.01	30
10.00	30
10.01	30
18.00	43
18.01	65
19.00	90
19.01	90
24.00	30

Supporting Figures

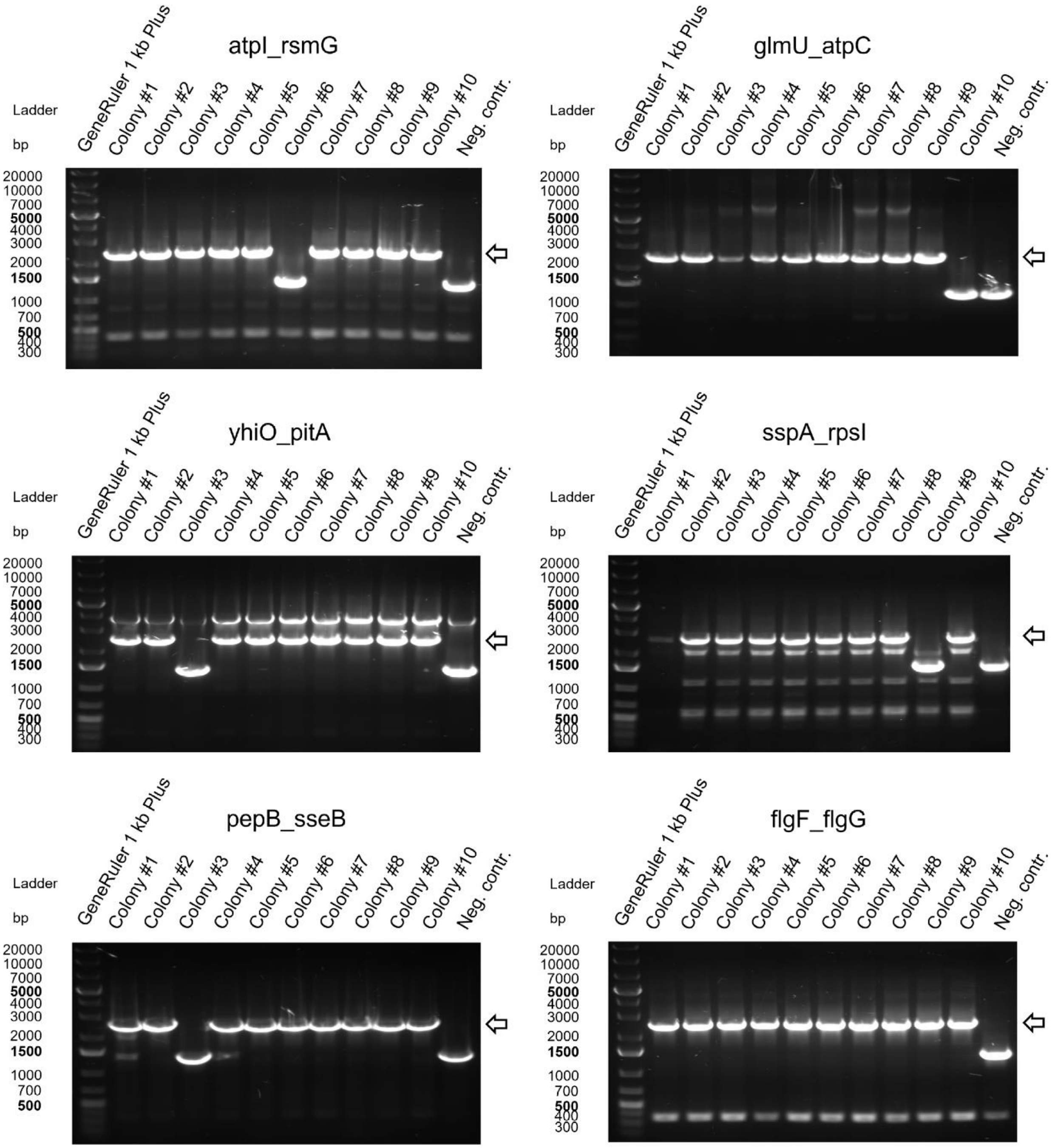

Figure S1: Agarose gels stained with MIDORI Green Advance under UV light. Colony PCR results after chromosomal integration of *mCherry* into different intergenic loci (*E. coli* BL21(DE3)). The amplicon lengths indicating successful integration events are marked by an arrow.

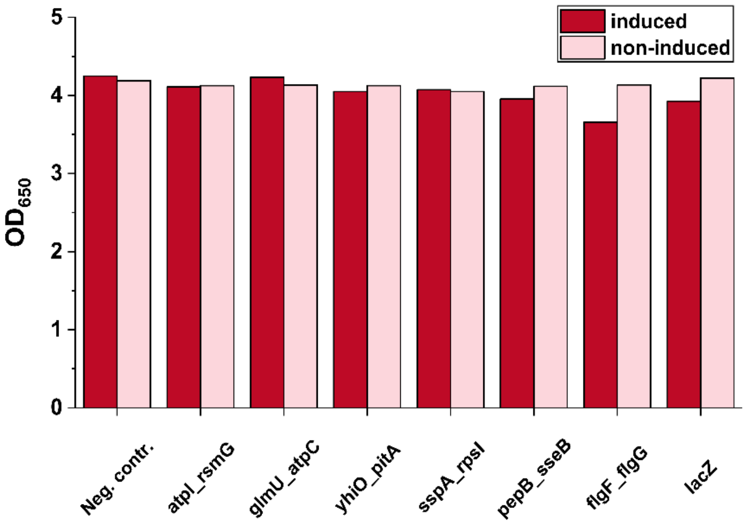

Figure S2. Impact of chromosomal integration and expression of *mCherry* on cell growth of *E. coli* BL21(DE3). *E. coli* BL21(DE3) without integrated genes was used as a negative control. Induced (dark red) and non-induced, basal (light red) expression were compared.

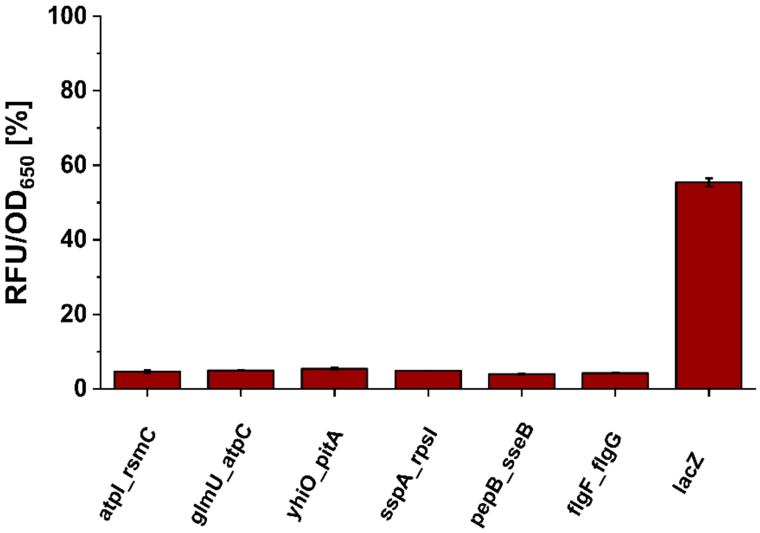

Figure S3. Non-induced (leaky) expression of *mCherry* after chromosomal integration into different genomic loci in *E. coli* BL21(DE3). Relative fluorescence was calculated in relation to induced expression after chromosomal integration into *lacZ* locus shown in Figure 2 in the main manuscript.

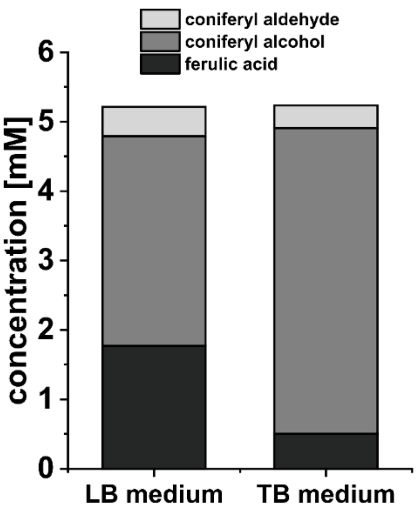

Figure S4: Medium comparison. Lysogeny broth (LB) and terrific broth (TB) were tested for conversion of 5 mM ferulic acid **1** by growing *E. coli* W3110(T7) cells harboring pETDuet_*Zm*CCR_*Pc*4CL. Concentrations of ferulic acid **1**, coniferyl aldehyde **3**, and coniferyl alcohol **4** were quantified 18 h after substrate addition via HPLC.

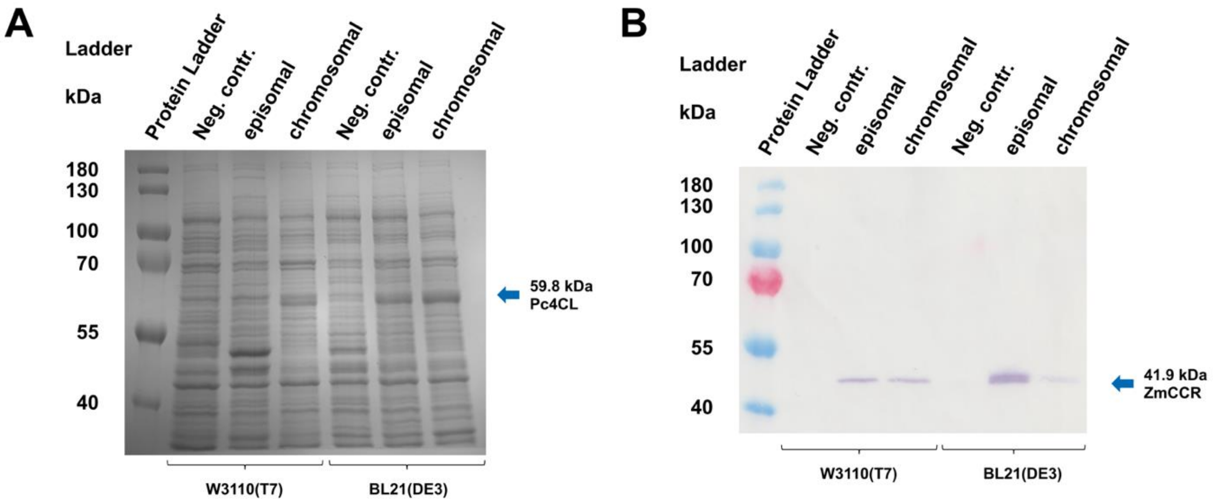

Figure S5: SDS-PAGE (A) and Western Blot (B) of whole cells 18 h after addition of 5 mM ferulic acid **1**. Expression of chromosomally integrated genes was compared to episomal expression using a pETDuet-1 vector. *E. coli* strains harboring no copy of heterologous genes were used as negative controls.

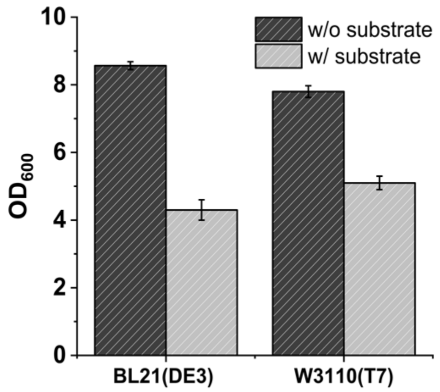

Figure S6: Impact of substrate addition on cell growth of different *E. coli* harboring chromosomally integrated genes encoding *Pc*4CL and *Zm*CCR. Optical density at 600 nm wavelength (OD_{600}) was measured 18 h after addition of 5 mM ferulic acid **1** (dissolved in DMSO). The same amount of DMSO was added to the samples without substrate to ensure comparability. Means and standard deviations were calculated from three biological replicates.

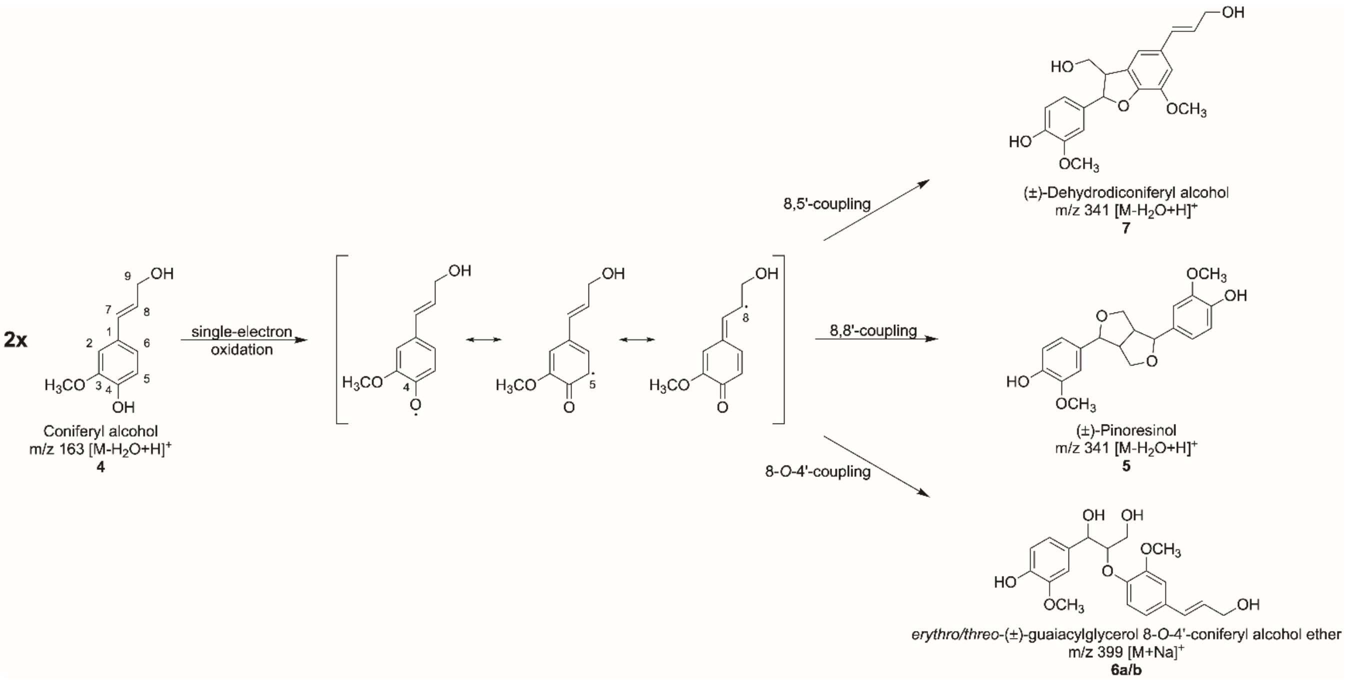

Figure S7: Oxidative radical coupling products of coniferyl alcohol **4**. Pinoresinol **5** originates from the 8,8'-coupling of two monomer radicals. The direction of approach determines which of the two enantiomers of **5** is formed (*si* face recombination results in (+)-pinoresinol, *re* face in (-)-pinoresinol). Thus, a racemic mixture is formed in the absence of dirigent proteins. m/z of base peaks observed in TIC(+) (LC/MS) is annotated to the compounds.

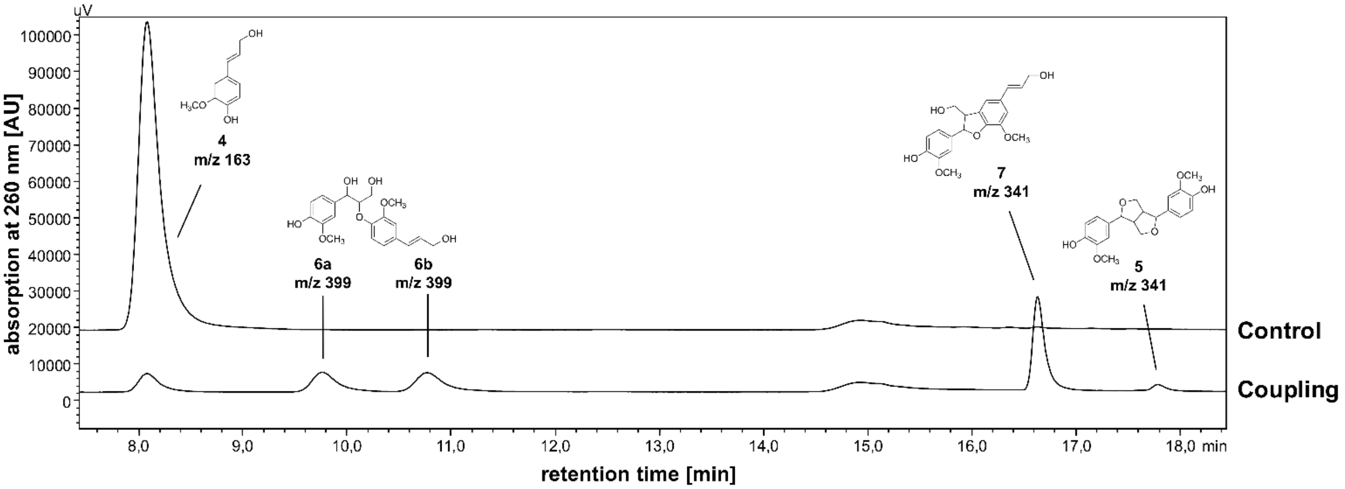

Figure S8: HPLC chromatogram for identification of oxidative coupling products of coniferyl alcohol **4**. Oxidative coupling was performed chemically by using $FeCl_3$ and resulted in the formation of pinoresinol **5**, *erythro*/*threo*-(±)-guaiacylglycerol 8-*O*-4'-coniferyl alcohol ethers **6a/b**, and dehydrodiconiferyl alcohol **7**. Control without addition of $FeCl_3$ shows no coupling products. Products were identified based on m/z according to our previous study (7). m/z of base peaks observed in the corresponding LC/MS TIC(+) are annotated.

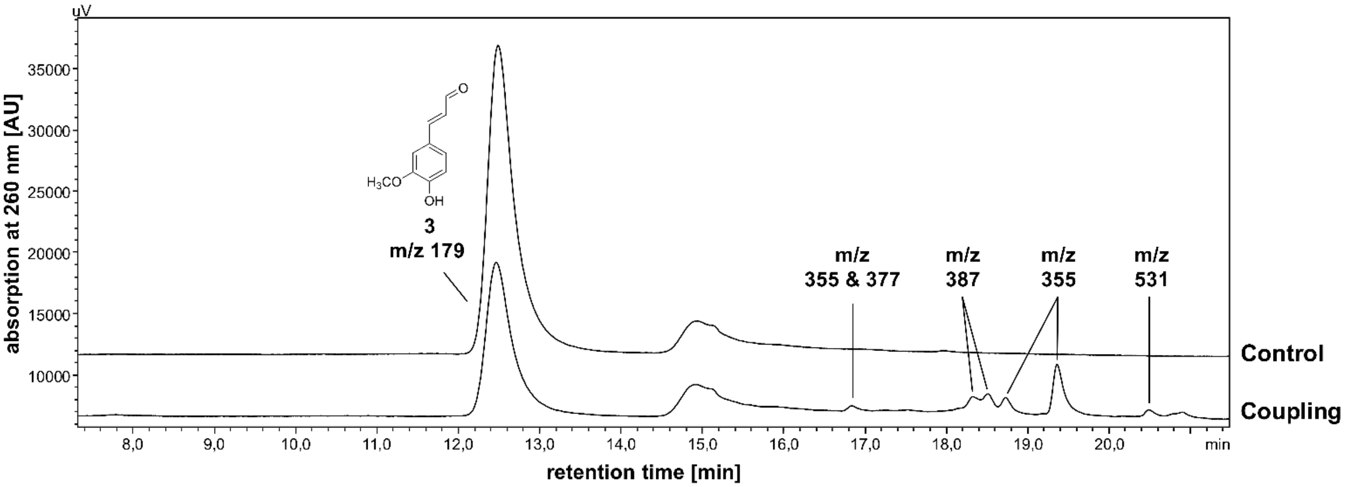

Figure S9: HPLC chromatogram for identification of oxidative coupling products of coniferyl aldehyde **3**. Oxidative coupling was performed chemically by using $FeCl_3$. Control without addition of $FeCl_3$ shows no coupling products. m/z of base peaks observed in the corresponding LC/MS TIC(+) are annotated.

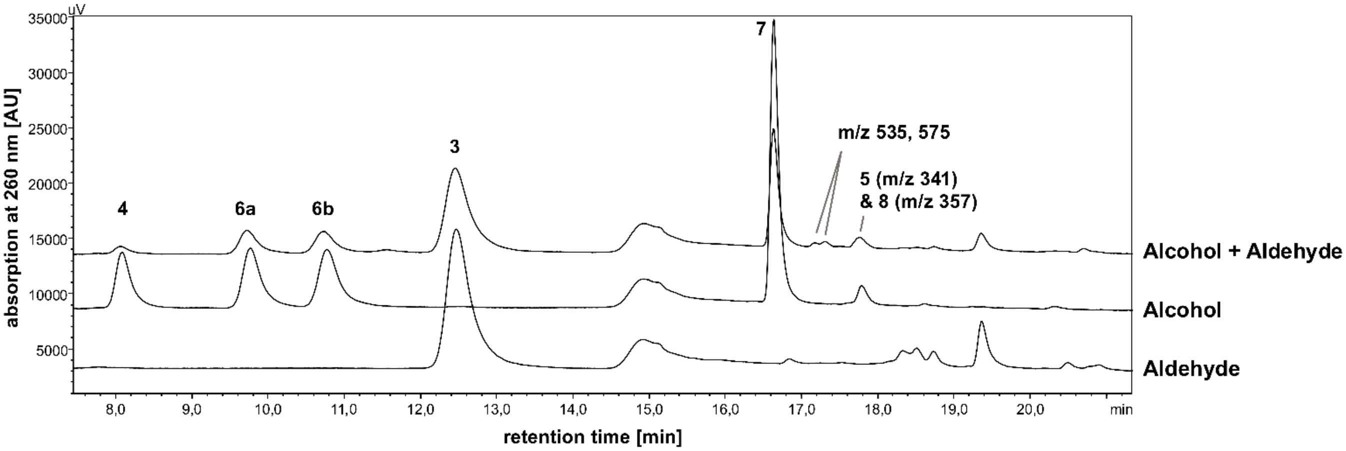

Figure S10: Comparison of HPLC chromatograms after $FeCl_3$-mediated coupling of coniferyl aldehyde **3** and coniferyl alcohol **4** performed separately (cf. Figures S8 & S9) as well as in the same reaction tube in order to identify cross-coupling products. m/z of base peaks observed in the corresponding LC/MS TIC(+) are annotated.

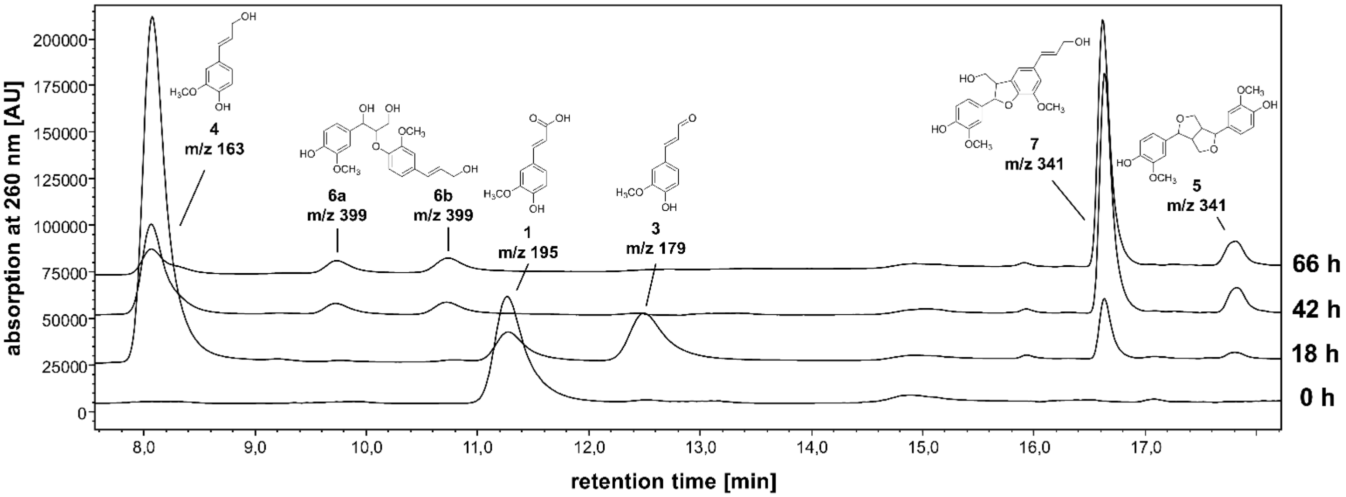

Figure S11: Comparison of HPLC chromatograms at different time points (0 h, 18 h, 42 h, 66 h after substrate addition) during conversion of 5 mM ferulic acid **1** to pinoresinol **5** (cf. Figure 4). The enzymatic cascade proceeds via coniferyl aldehyde **3** and alcohol **4**. Laccase-catalyzed radical C-C-coupling resulted in the formation of pinoresinol **5**, *erythro*/*threo*-(±)-guaiacylglycerol 8-*O*-4'-coniferyl alcohol ethers **6a/b**, and dehydrodiconiferyl alcohol **7**. m/z of base peaks observed in the corresponding LC/MS TIC(+) are annotated.

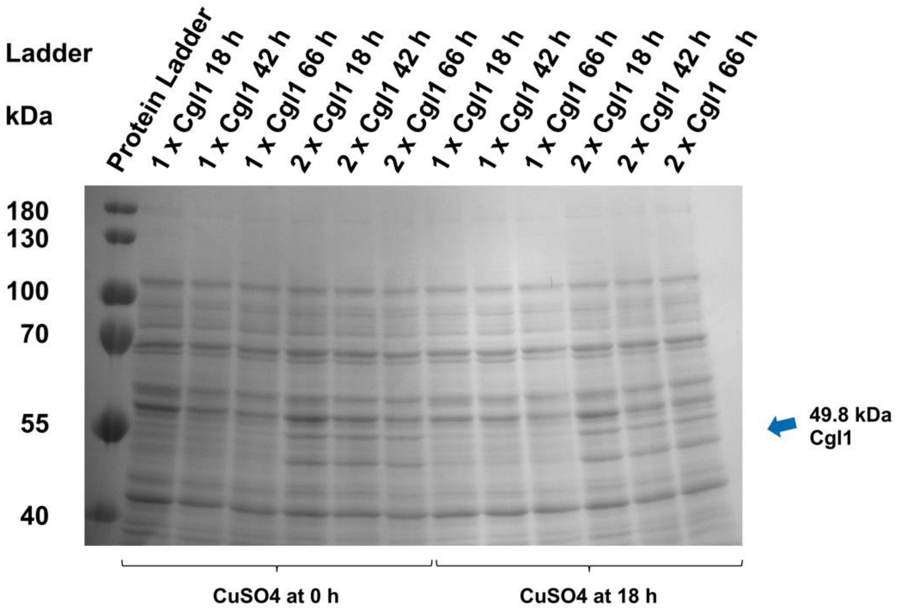

Figure S12: SDS-PAGE of whole cells 18 h, 42 h, and 66 h after addition of 5 mM ferulic acid **1**. $CuSO_4$ addition at time of induction (0 h) was compared to 18 hours after induction. Additionally, different copy numbers of *cgl1* (one or two copies) were compared (cf. Figure 5).

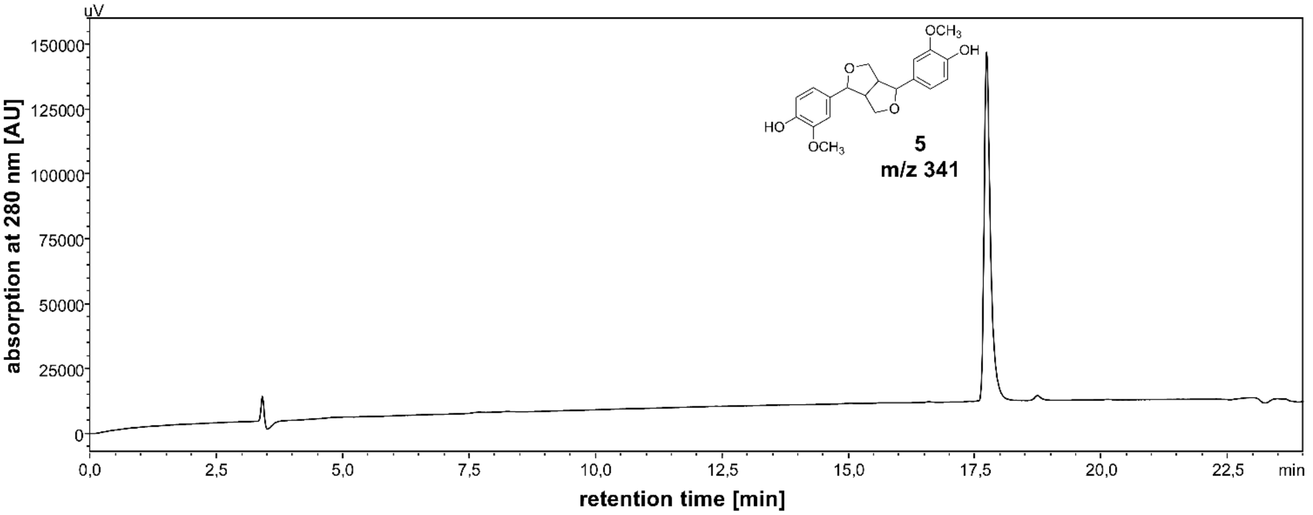

Figure S13: HPLC chromatogram of isolated pinoresinol **5** for determination of purity. m/z of the base peak observed in the corresponding LC/MS TIC(+) is annotated.

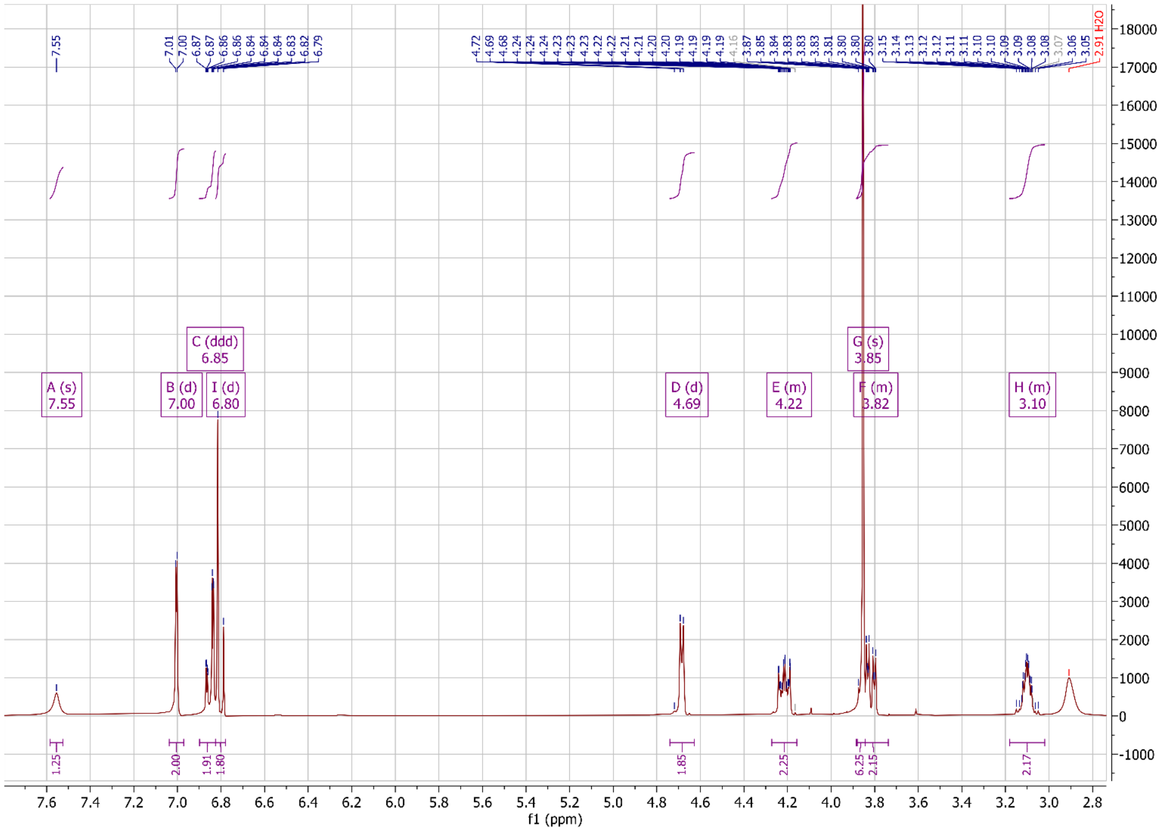

Figure S14: ^{1}H-NMR (300 MHz, Acetone) δ 7.55 (s, 1H), 7.00 (d, J = 1.9 Hz, 2H), 6.85 (ddd, J = 8.1, 1.9, 0.6 Hz, 2H), 6.80 (d, J = 8.0 Hz, 2H), 4.69 (d, J = 4.3 Hz, 2H), 4.27 – 4.16 (m, 2H), 3.85 (s, 6H), 3.88 – 3.74 (m, 2H), 3.18 – 3.02 (m, 2H).

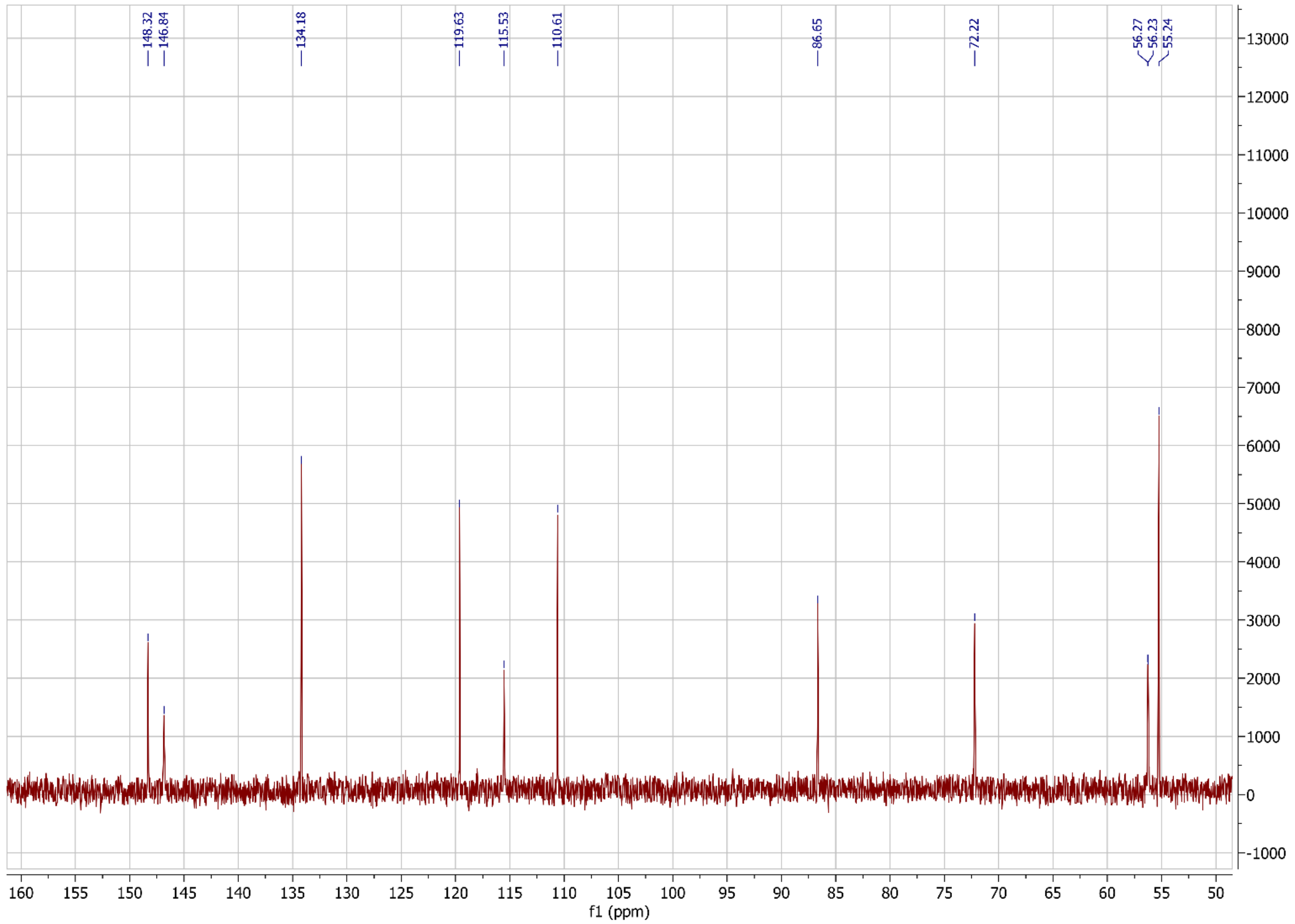

Figure S15: ^{13}C-NMR (75 MHz, Acetone) δ 206.28, 148.32, 146.84, 134.18, 119.63, 115.53, 110.61, 86.65, 72.22, 56.27, 56.23, 55.24.

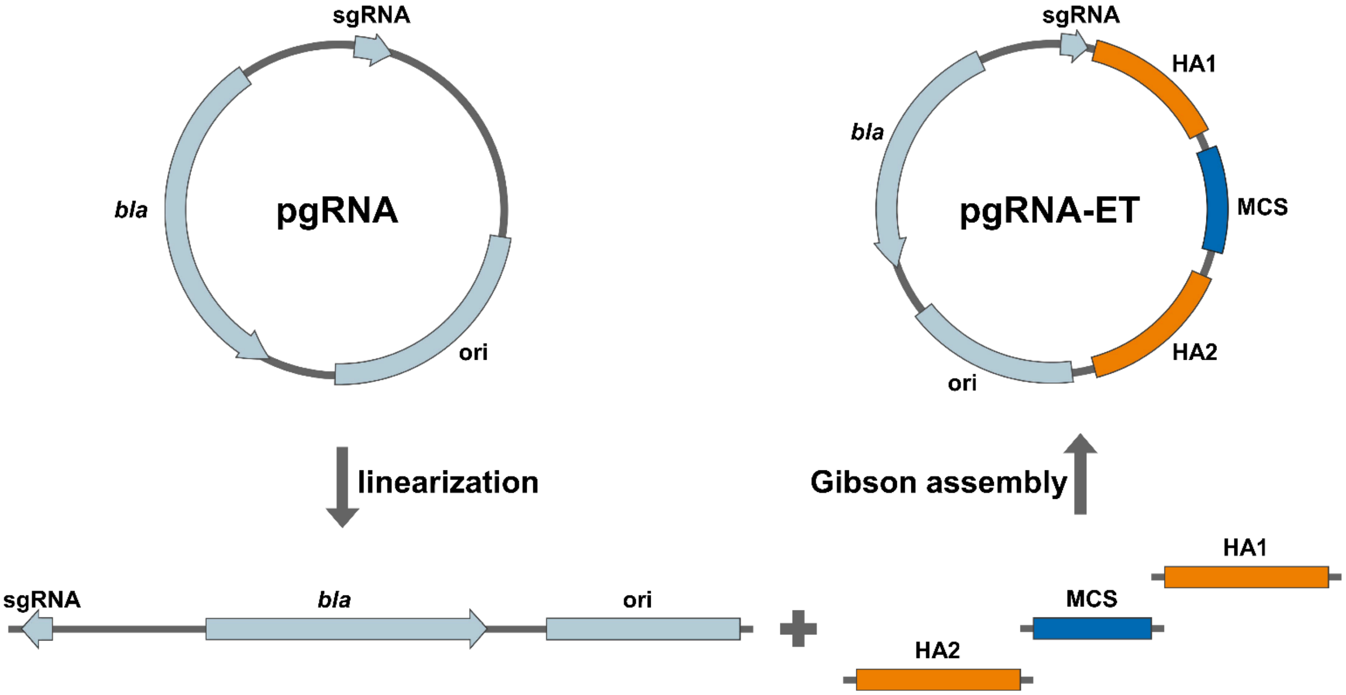

Figure S16. Construction of the plasmid series pgRNA-ET. The plasmid pgRNA (left) contains a locus-specific single-guide RNA (sgRNA) under control of a constitutive promoter, a ColE1 origin of replication (ori) as well as a gene encoding the β-lactamase (*bla*) for ampicillin resistance (3). PCR was used to linearize the plasmid and introduce overlaps for Gibson assembly. Locus-specific homology arms of ~500 bp length (HA1 & HA2) for homologous recombination during the chromosomal integration process were amplified from *E. coli*'s genome. The expression cassette containing the multiple cloning site (MCS) originated from pET-28a(+). All four fragments were fused by Gibson assembly to result in the pgRNA-ET plasmid series.

References

1. Luelf UJ, Böhmer LM, Li S, Urlacher VB. Effect of chromosomal integration on catalytic performance of a multi-component P450 system in Escherichia coli. Biotechnol Bioeng. 2023;120(7):1762-72.
2. Li Q, Sun B, Chen J, Zhang Y, Jiang Y, Yang S. A modified pCas/pTargetF system for CRISPR-Cas9-assisted genome editing in *Escherichia coli*. Acta Biochimica et Biophysica Sinica. 2021;53(5):620-7.
3. Qi LS, Larson MH, Gilbert LA, Doudna JA, Weissman JS, Arkin AP, Lim WA. Repurposing CRISPR as an RNA-Guided Platform for Sequence-Specific Control of Gene Expression. Cell. 2013;152(5):1173-83.
4. Jansen F, Gillessen B, Mueller F, Commandeur U, Fischer R, Kreuzaler F. Metabolic engineering for *p*-coumaryl alcohol production in *Escherichia coli* by introducing an artificial phenylpropanoid pathway. Biotechnol Appl Biochem. 2014;61(6):646-54.
5. Ricklefs E, Girhard M, Koschorreck K, Smit MS, Urlacher VB. Two-Step One-Pot Synthesis of Pinoresinol from Eugenol in an Enzymatic Cascade. ChemCatChem. 2015;7(12):1857-64.
6. Seo J-H, Baek S-W, Lee J, Park J-B. Engineering *Escherichia coli* BL21 Genome to Improve the Heptanoic Acid Tolerance by using CRISPR-Cas9 System. Biotechnology and Bioprocess Engineering. 2017;22(3):231-8.
7. Decembrino D, Girhard M, Urlacher VB. Use of Copper as a Trigger for the *in Vivo* Activity of *E. coli* Laccase CueO: A Simple Tool for Biosynthetic Purposes. ChemBioChem. 2021;22(8):1470-9.
